# Electrochemical N-Formylation of Amines: Mechanistic Insights and Sustainable Synthesis of Formamides via a Methylisocyanide Intermediate

**DOI:** 10.1021/jacs.4c16725

**Published:** 2025-03-12

**Authors:** Pim J. L. Broersen, Lars Wielhouwer, Gadi Rothenberg, Amanda C. Garcia

**Affiliations:** Van‘t Hoff Institute for Molecular Sciences, University of Amsterdam, Science Park 904, 1098 XH Amsterdam, The Netherlands

## Abstract

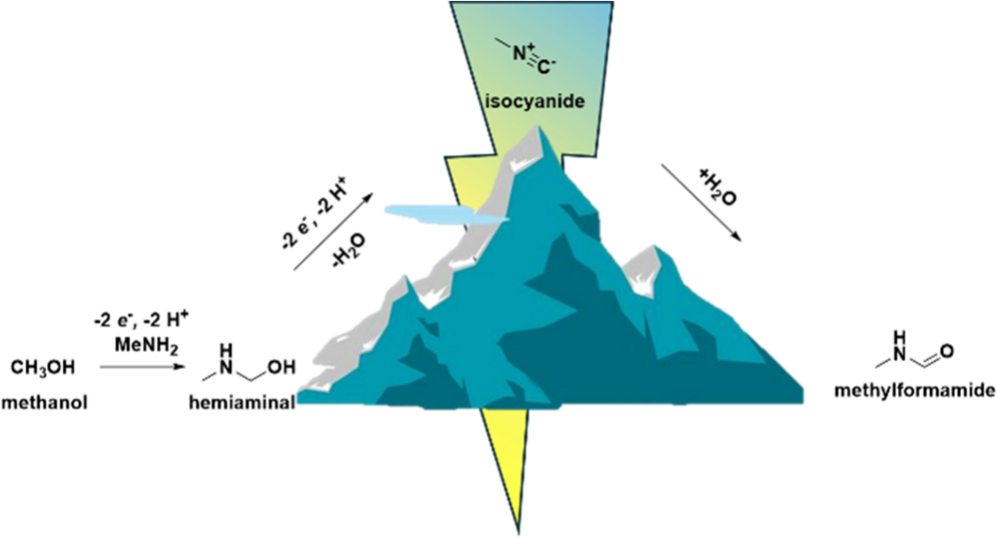

Electrochemical methodologies offer a transformative approach to sustainable chemical synthesis by enabling precise, energy-efficient transformations. Here, we report the selective electrochemical N-formylation of methylamine using methanol as both reagent and solvent, facilitated by a simple glassy carbon electrode. Under optimized conditions, we achieve a faradaic efficiency (FE) of 34% for methylformamide synthesis in a neutral NaClO_4_ electrolyte. Mechanistic insights from in situ Fourier-transform infrared spectroscopy (FTIR) and complementary synthetic experiments reveal two distinct reaction pathways: the direct oxidation of a hemiaminal intermediate and a novel route involving the formation of methylisocyanide, which subsequently hydrates to yield methylformamide. The presence of methylisocyanide was confirmed through mass spectrometry analysis following a successful Ugi multicomponent reaction, demonstrating the ability to safely utilize reactive intermediates within an electrochemical framework. This work underscores the potential of electrosynthesis to unlock metal-free, sustainable pathways to produce value-added nitrogen-containing compounds, paving the way for greener approaches in chemical manufacturing and catalysis.

## Introduction

Today’s chemical industry relies on thermochemical processes and fossil-carbon based feedstocks.^1^ To comply with the 2015 Paris Agreement,^2^ a transition to alternative feedstocks is needed. Electrochemistry presents a promising approach in the transition to more sustainable industrial processes.^3^ Among many possible electrosynthetic reactions, C–N bond formation, fundamental to the production of amides, amines, and other nitrogen-containing compounds, is crucial for making many pharmaceuticals, agrochemicals and materials.^4^ In recent years, different methods were developed to synthesize small amides, specifically formamides and acetamides, through electrocatalytic means.^5−12^

Particularly, amides, such as formamides and acetamides serve as solvents, catalysts, and intermediates in both laboratory and industrial processes. For example, dimethylformamide (DMF) is one the most widely used amide solvent in the EU,^13^ although dimethylacetamide is preferred due to its lower chronic toxicity.^14^ More complex formamides are used in various niche applications,^15−17^ but their key value is as precursors to isocyanides.^18−20^ These foul-smelling, yet highly useful compounds are obtained through treating the formamides with strong dehydration agents. Traditional routes to produce these compounds are energy-intensive and heavily reliant on fossil-carbon resources, underscoring the need for sustainable alternatives. In this context, electrocatalytic methods present a promising approach, offering a cleaner, potentially carbon-neutral pathway to C–N bond formation.

**Figure 1:**
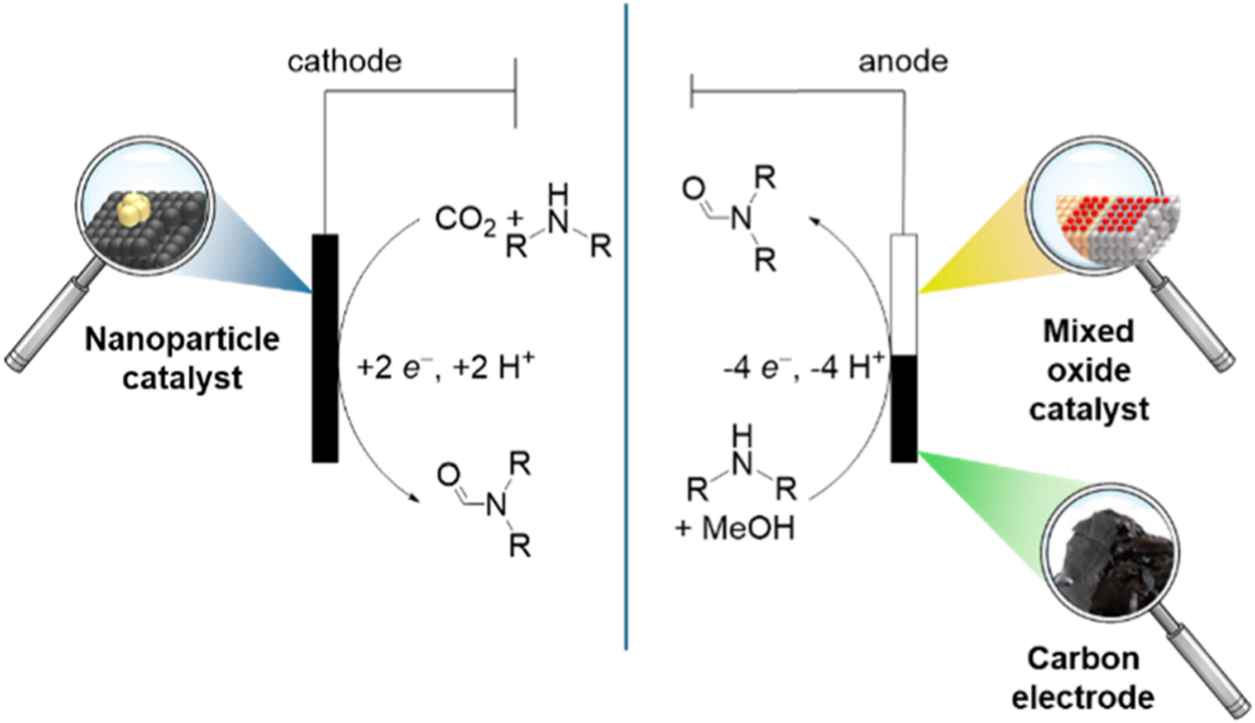
Schematic representation of the reductive (left) and oxidative (right) electrocatalytic routes to amides.

The electrochemical synthesis of small amides can be divided in two different routes (Figure 1): combined reduction of CO_2_ and a nitrogen source^6,7,12^ or combined oxidation of methanol and a nitrogen source.^10,11^ The former allows for amide synthesis from waste streams, especially if the nitrogen source is a nitrite or a nitrate. However, the reductive route often requires complicated electrode materials with platinum group metals,^12^ limiting its practicality. In contrast, studies on the oxidative route are relatively scarce. Zhang and co-workers have explored this pathway, demonstrating its feasibility using ammonia as the amine source.^9−11^ These studies were performed with different types of electrodes, namely Pt, Pr doped MnO_2_ and boron doped diamond (BDD). Faradaic efficiencies ranged from 23 to 41% for the reaction. Recently, another study reported the successful application of dimethylamine as the starting material, achieving an FE of 47% for dimethylformamide on a W/Ni based electrode.^21^

Here we report the synthesis of N-methylformamide from methylamine (MeNH_2_) and methanol using a simple glassy carbon electrode, with a maximum FE of 34%. Importantly, we also quantify accurately the formaldehyde intermediate. By using in situ Fourier-transform infrared (FTIR) spectroscopy, and by running control and further synthetic experiments, we confirmed that methylisocyanide is an important intermediate in this reaction. Additionally, we ran an Ugi reaction with the isocyanide intermediate after electrolysis. Our results show two important findings: First, that small formamides can be synthesized using metal-free electrode materials. Second, that using electrochemistry one can run isocyanide-based multicomponent reactions with hazardous chemicals such as formaldehyde and methylisocyanide in a safe and controlled manner.

By offering an accessible, metal-free pathway to valuable formamide compounds, our method exemplifies a promising shift toward greener and more efficient C–N bond formation techniques. This study shows a novel route for sustainable amide synthesis and open opportunities for electrochemically driven multicomponent reactions, underscoring the vast potential of electrosynthesis in advancing the field of sustainable chemistry.

## Experimental Section

All water used in this research was purified using a Milli-Q Millipore system with a total organic carbon content lower than 3 ppb and a resistivity higher than 18 MΩ·cm at room temperature. Prior to electrochemical experiments, all glassware was stored overnight in a large beaker containing an aqueous 1 g L^–1^ KMnO_4_ (VWR chemicals, GPR RECTAPUR) and 0.5 M H_2_SO_4_ (Sigma-Aldrich, ACS reagent) solution. Before use, the KMnO_4_ solution was removed from the beaker and the glassware in the beaker was rinsed three times with Milli-Q water. The glassware was then immersed in a dilute aqueous H_2_SO_4_/H_2_O_2_ (1:0.3 M) solution (so-called “piranha solution”) to further oxidize the leftover organic and KMnO_4_ residue. After removing the piranha solution and rinsing the glassware, the glassware was boiled in fresh Milli-Q water three times before use.

All electrochemical experiments were performed at room temperature. Unless stated otherwise, chemicals were purchased from commercial companies and used as received.

For electrochemical characterization and electrolysis, a circular glassy carbon (GC, MaTecK, ø = 6 mm) electrode was used. Prior to each measurement, the glassy carbon was mechanically polished with a 3.0 μm and a 1.0 μm diamond suspension (Buehler) on a MicroCloth polishing cloth (Buehler). For both CV and bulk electrolysis graphite (MaTecK, ø = 6 mm, *l* = 90 mm) and a saturated calomel electrode (SCE, Gamry) were used as counter and reference electrodes (CE and RE), respectively. Prior to all measurements, the electrolyte was degassed by bubbling with N_2_ gas for 15 min.

### Bulk Electrolysis

The preparative scale reactions were performed in a two-compartment electrochemical glass cell configuration divided by a Nafion-117 membrane (FuelCellstore). Each compartment was filled with 10 mL MeOH (Sigma-Aldrich, spectrophotometric grade, >99.9%), as well as the appropriate amount of MeNH_2_ (Sigma-Aldrich, 40% aq. solution) and 0.1 M supporting electrolyte (NaOH, NaClO_4_, H_2_SO_4_ (0.05 M)). The catholyte compartment contained a graphite CE (MaTecK, ø = 6 mm, *l* = 90 mm). The anolyte compartment contained an SCE (Gamry) and GC for the RE and WE, respectively. The solution was purged with N_2_ gas (Linde Gas, 99.999%) for 15 min and subsequently kept under inert atmosphere by passing a constant N_2_ stream over it. Electrolysis was performed at the desired potential with both an unstirred catholyte and anolyte compartment, using an Autolab Potentiostat (Metrohm M204). For the optimization studies, electrolysis was run for 1 h. At the end point of the reaction, the reaction mixture was analyzed for the presence of formaldehyde and formamide.

### High Performance Liquid Chromatography Analysis

The formamide products were analyzed using a high-performance liquid chromatography (HPLC) Agilent 1260 Infinity II system equipped with an Agilent Technologies Inc. Aminex HPX 87-H column (300 mm × 7.8 mm) and a RID and VWD detector. An isocratic chromatography method was used with an eluent of 5 mM H_2_SO_4_ in water and a column temperature of 65 °C, using a flow rate of 1 mL min^–1^. The formamide product yield was calculated by fitting the results on a predetermined regression line (Figure S1).

### Procedure for Formaldehyde Analysis

To analyze the formaldehyde produced in this reaction we used our modified dinitrophenylhydrazine (DNPH) derivation method.^22^ A 50 μL aliquot was taken from the reaction mixture and diluted 30 times in water. Of this diluted sample, 50 μL was added to a mixture of 400 μL of acetonitrile and 500 μL of water. Then, 50 μL of a 0.3 wt % solution of DNPH (TCI chemicals, >98.0%) in phosphoric acid (Alfa Aesar, 85% aq. solution) was added. The sample was heated to 75 °C for 35 min and subsequently analyzed via HPLC. Analysis was done on an Agilent 1260 Infinity II system equipped with an Agilent Technologies Inc. Poroshell 120 EC-C18 column (150 mm × 3 mm, 2.7 μm) and a RID and VWD detector. The column temperature was maintained at 35 °C. The mobile phase was acetonitrile/5 mM H_2_SO_4_, which was run in a gradient from 20/80 v/v to 50/50 v/v after 3 min and 90/10 v/v after 8 min at a flow rate of 1 mL min^–1^. The formaldehyde derivative was detected via UV at 350 nm and the final concentration was determined by fitting the results to a predetermined regression line (Figure S2).

### Procedure for Cyanide Analysis

To analyze any cyanide that could have formed during the reaction we adapted a previously published derivation method.^23,24^ A phosphate buffer (pH 6.8) was prepared out of potassium dihydrogen phosphate (Merck Life Science, 6.80 g, 0.050 mol, 2.00 equiv), KOH (MaTecK, 99+%) (1.40 g, 0.0250 mol, 1.00 equiv) and NaBO_2_.4H_2_O (Fisher Scientific, 98.5%) (3.45 g, 0.0250 mol, 1.00 equiv) in 500 mL water. This buffer was used to make a taurine and a naphthalene-2,3-dicarboxaldehyde (NDA) solution. The taurine solution was made by diluting taurine (Fluorochem Ireland) (313 mg, 2.5 mmol) in 50 mL of buffer. The NDA solution was prepared by dissolving NDA (TCI chemicals, >99.0%) (36.8 mg, 0.200 mmol) in 40 mL methanol. Afterward 60 mL buffer was added to this solution. Separately, a 400 ppm KCN stock solution was prepared by dissolving KCN (Fluka, >97.0%) (50 mg, 0.0075 mol) in 50 mL 0.01 M NaOH solution.

The analysis method was tested by diluting 10 μL of the KCN stock was diluted in 990 μL 0.001 M NaOH solution. 125 μL of this mixture was added to a vial together with 375 μL 0.001 M NaOH in methanol, 100 μL taurine solution, and 100 μL NDA solution. This was then left in the dark for 30 min at room temperature and afterward analyzed using HPLC. Analysis was done on an Agilent Technologies Inc. Poroshell 1260 Infinity II system equipped with an Agilent Technologies Inc. Poroshell 120 EC–C18 column (150 mm × 3 mm, 2.7 μm) and an RID and VWD detector. The column temperature was maintained at 35 °C. The mobile phase was acetonitrile/5 mM H_2_SO_4_, which was run in a gradient from 10/90 v/v to 80/20 v/v after 25 min at a flow rate of 0.5 mL min^–1^. The cyanide NDA and taurine derivative was detected via UV at 418 nm with a retention time of 12.80 min.

The influence of methylamine with NDA and cyanide was tested as well. A 0.5 M methylamine solution was prepared by adding 15 μL of the 40% methylamine solution to 1000 μL of the 0.001 M NaOH solution. Instead of 375 μL 0.001 M NaOH solution, 200 μL of the methylamine solution was added and 175 μL of the 0.001 M NaOH solution was added. The rest of the sample preparation and analysis was identical to the KCN test described above. The results showed that methylamine also reacted with CN^–^ and NDA. This compound was also detected via UV at 418 nm with a retention time of 19.40 min. To confirm that this was indeed the NDA methylamine derivative the compound was also analyzed using LC-MS which showed a peak with a mass of 207.02, which corresponds to the mass of the protonated molecule. LC-MS was performed on a Shim-pack GIST-HP C18-AQ 3 μm; 100 mm × 4.6 mm (I.D) column, with 0.1% aq. HCOOH/0.1% HCOOH in acetonitrile as the mobile phase. This was run from 95/5 v/v (5 min hold), to 5/95 v/v after 3 min (2 min hold), back down to 95/5 v/v in 1.5 min. (3.5 min hold). The compounds were detected by UV–vis from 200 to 600 nm, The corresponding electrospray ionization (ESI-Pos) spectra were collected on a HR-ToFBruker Daltonik GmbH (Bremen, Germany) Impact II.

Finally, reaction mixtures were analyzed with this adapted method. An aliquot of the reaction mixture was taken and diluted 10 times in methanol. 125 μL of this mixture was added to a vial together with 375 μL 0.001 M NaOH in methanol, 100 μL taurine solution, and 100 μL NDA solution. This was then left in the dark for 30 min at room temperature and afterward analyzed using HPLC with the same method. Neither cyanide derivative was found.

### In Situ Fourier Transform Infrared Spectroscopy

To investigate the mechanistic details of the reaction, we performed in situ Fourier Transform Infrared spectroscopy (FTIR) for the methanol oxidation processes. The experiments were carried out in a Bruker Vertex 80-V IR spectrometer equipped with a liquid nitrogen cooled MCT detector. A Veemax III (PIKE Technologies) was positioned in the spectrometer, wherein a homemade three electrode spectroelectrochemical cell with a CaF_2_ prism attached to the bottom was placed. We performed the FTIR experiments in all electrolytes used in bulk electrolysis (0.1 M NaOH, 0.1 M NaClO_4_ and 0.05 M H_2_SO_4_) with and without the addition of 0.5 M MeNH_2_. Approximately 5 mL of each mixture was used for all experiments. Glassy carbon disc (MaTeck, ø = 5 mm) was used as WE, while RHE and Pt wire were used as reference and counter electrodes, respectively.

To convert the measurements from RHE to the SCE scale, the following formula can be used.
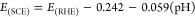
1

We assume that the pH of the 0.5 M MeNH_2_ in methanol in all electrolytes is approximately 12 (pH of 0.5 M MeNH_2_ in H_2_O). This assumption is not entirely correct due to the fact that methanol is a different solvent than water, but previous research on the behavior of bases in methanol shows that this is a good approximation, due to the p*K*_a_ of methanol and water being similar.^25^ This means that the spectra measured at 1.0 V vs RHE refer to a potential of 0.05 V vs SCE.

Prior to the measurements, the solution was purged with argon for 15 min and subsequently kept under inert atmosphere. The WE was pressed against the CaF_2_ prism to obtain a thin film configuration. Spectra were collected over a potential range in 0.1 V increments from 1.0 to 4.4 V vs RHE, or until oxygen evolution deteriorated the quality of the data. FTIR spectra were collected in a range of 4000–1000 cm^–1^ at a resolution of 8 cm^–1^ for 100 scans. The spectra are presented as absorbance, according to *A* = −log(*R*/*R*_0_), where *R* and *R*_0_ are the reflectance corresponding to the single beam spectra obtained at the sample and reference potentials, respectively. In these configuration, negative bands (pointing down) correspond to the species that were present on or near the electrode surface at the reference potential and that are “consumed” at the sample potential. Positive bands (pointing up) correspond to the formation of species at the sample potential. All the spectro-electrochemical experiments were performed at room temperature.

## Results and Discussion

**Scheme 1:**
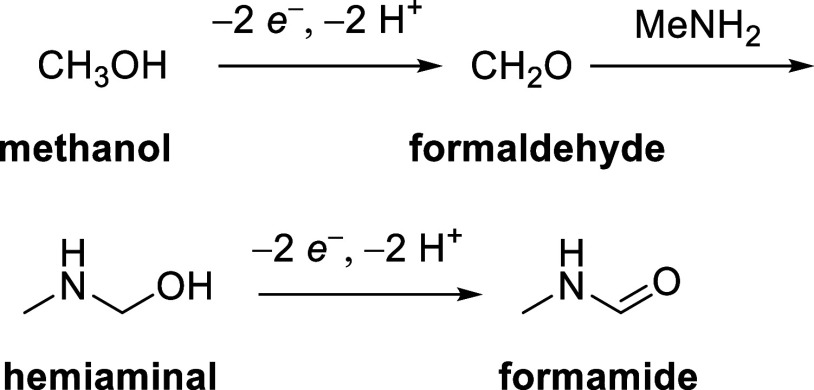
Electrochemical Oxidation of Methanol to N-Methylformamide with MeNH_2_

We started by studying the electrochemical N-formylation with MeNH_2_ using methanol as both solvent and reagent. In this system, methanol is first electrochemically oxidized to formaldehyde, which in turn reacts with the amine to give first the hemiaminal, followed by its oxidation the amide (Scheme 1).

**Figure 2:**
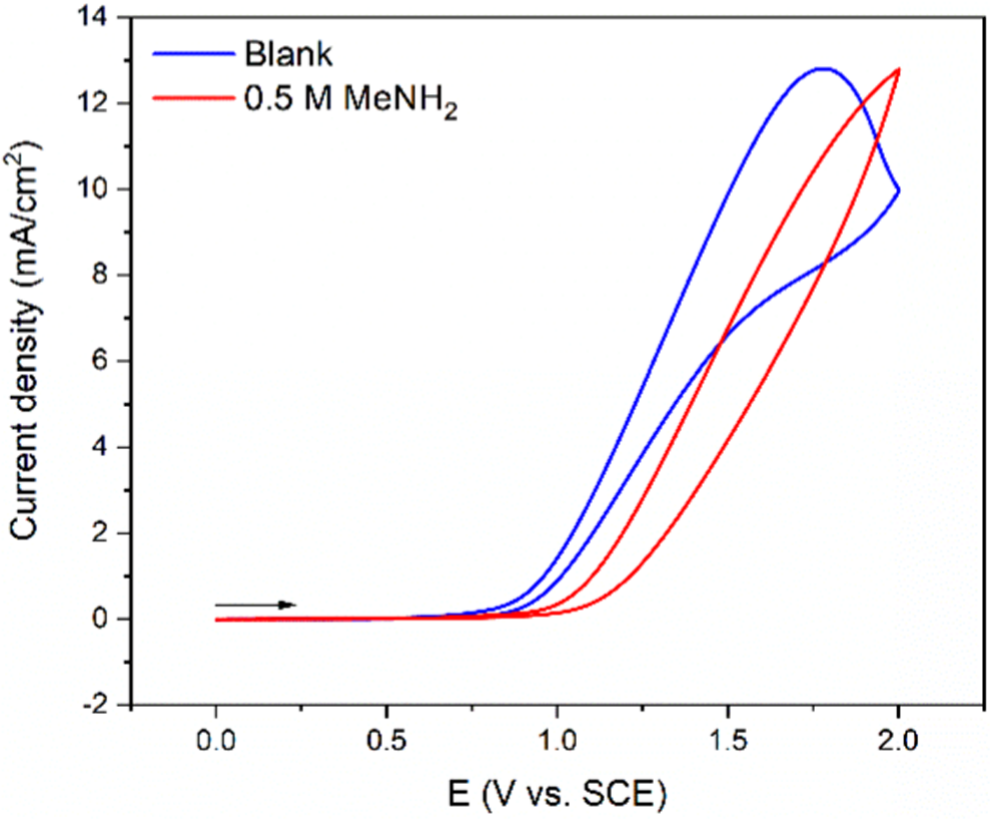
Cyclic voltammograms of a glassy carbon electrode in 0.1 M NaOH in methanol (blue curve) and with addition of 0.5 M MeNH_2_ (red curve) (scan rate = 100 mV s^–1^; arrow indicates the scan direction).

Previously, we showed that methanol oxidation proceeds in the presence of a base, which activates the methanol.^26^ Thus, we began by using 0.1 M NaOH as the supporting electrolyte. Figure 2 compares the cyclic voltammogram of glassy carbon electrode in pure 0.1 M NaOH in methanol (blue line) and with the addition of 0.5 M MeNH_2_. In absence of amine (MeNH_2_) the onset potential for oxidation of methanol is at approximately 0.7 V vs SCE, whereas the addition of MeNH_2_ causes a shift of the onset to more positive potential (ca. 0.9 V vs SCE), suggesting the oxidation of hemiaminal species to amide happens at slight higher potential than the oxidation of methanol to formaldehyde. To further understand the reaction mechanism and product formation, we performed bulk electrolysis reactions under different conditions.

### Bulk Electrolysis

**Figure 3:**
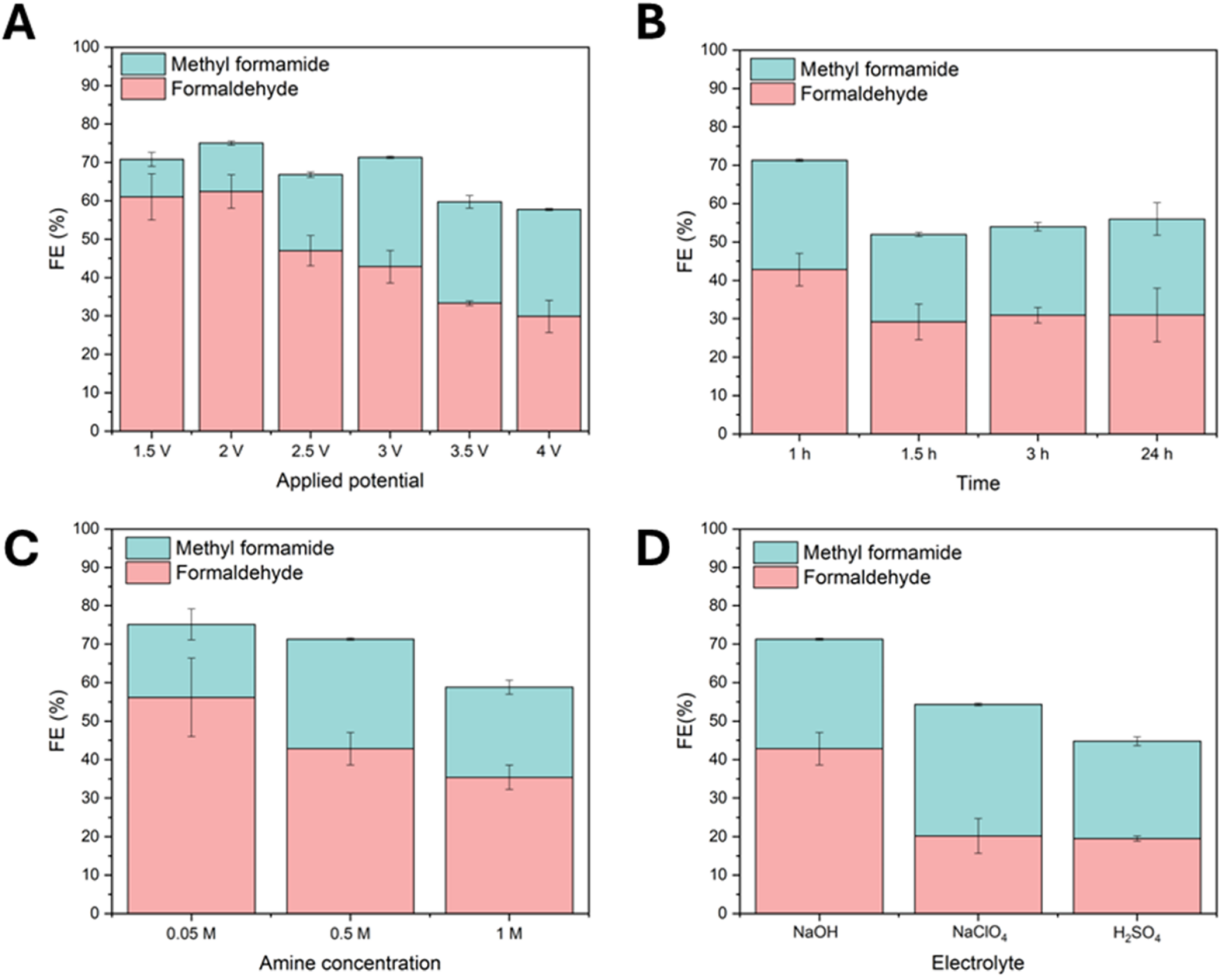
Faradaic efficiency optimization studies of N-methylformamide electrosynthesis varying the potential (A), time (B), amine concentration (C) and electrolyte (D). Standard conditions for the reaction were 0.5 M MeNH_2_, 0.1 M NaOH in methanol, using a GC WE, graphite CE applying a potential of 3.0 V vs SCE for 1 h in a divided cell setup. Variations to standard conditions are indicated in the graph.

The formamide electrosynthesis was optimized by varying several parameters such as potential, amine concentration and electrolyte (Figure 3). Earlier research by Zhang and co-workers showed that the amine concentration, pH and current density are crucial to obtaining a high FE.^9−11^ To study the role of the current density, we ran electrolysis reactions at potentials ranging from 1.5 to 4.0 V vs SCE. The results are shown in Figure 3A. There is a positive correlation between the applied potential and the formamide product, until 3 V vs SCE, where a maximum FE for methylformamide of 28.5% was reached, with a formaldehyde FE of 42.8%. At higher potentials, the FE for methylformamide drops. The FE for formaldehyde is inversely correlated with the applied potential. The lower FE for methylformamide at potentials higher than 3.0 V vs SCE likely reflects the lower availability of the formaldehyde intermediate at the electrode. The dropping FE for formaldehyde can either be through overoxidation of the formaldehyde to formate or CO_2_, or because of the oxygen evolution reaction (OER) that starts to dominate at higher potentials. However, we found only traces of formate in our HPLC samples, ruling out formate production as a major side reaction.

We also studied how the FE for methylformamide changed over time (Figure 3B). After 24 h, the formaldehyde FE dropped to 31% while the methylformamide FE dropped only slightly, to 25%, showing that the reaction rate remains relatively stable over this time frame.

The amine concentration was optimal at 0.5 M (Figure 3C), with higher and lower amine concentrations showing lower formamide FE, with 0.05 M MeNH_2_ resulting in 19% methylformamide FE and 1.0 M MeNH_2_ giving 23.4% FE. At lower amine concentrations a higher FE for formaldehyde was reached, while at higher concentration the FE for formaldehyde dropped. At lower amine concentration, less hemiaminal intermediate is formed, so less oxidation of the hemiaminal is possible and formaldehyde FE is higher. As the amine concentration is increased the amount of hemiaminal oxidation increases as well. The lower total FE at 1.0 M MeNH_2_ is likely due to more water oxidation, since methylamine is added as a 40% aqueous solution.

After optimization of the amine concentration, we studied the role of the pH in the reaction. Surprisingly, switching from a basic to a neutral or acidic environment caused a measurable, but not a large difference in methylformamide FE (28.5% for NaOH, 34.1% for NaClO_4_ and 19.5% for H_2_SO_4_). The FE for formaldehyde was highest with NaOH as the supporting electrolyte. Although we initially selected a basic environment to perform the reaction, the excess of MeNH_2_ can likely fulfill this role in the neutral or acidic electrolyte. The stronger basic environment of NaOH as the electrolyte can be important for the methanol activation toward formaldehyde, which explains the higher formaldehyde FE under these conditions. The optimum methylformamide FE of 34.1 ± 0.4% was achieved with a neutral electrolyte, namely 0.1 M NaClO_4_. Under these conditions, there is likely an optimum between methanol oxidation and subsequent hemiaminal oxidation to the desired product.

### Mechanistic Studies

The first step in studying the mechanism for this reaction is to show the involvement of formaldehyde as the intermediate in this reaction, formed through the oxidation of methanol. To do so we mixed formaldehyde (37 wt % in water) and methylamine (40 wt % in water) in equal molar ratios. 0.1 M NaClO_4_ was added as the supporting electrolyte. No methanol was added to the reaction to exclude methanol oxidation. Electrolysis was performed at 3.0 V vs SCE for 1 h. Methylformamide was formed with an FE of 24%. Because the starting materials were both added as aqueous solutions, OER is expected as a major side reactions, especially at this potential. However, this is slightly counteracted by the fact that both starting materials are added in equimolar ratios, allowing for the maximum amount of hemiaminal formation.

**Figure 4:**
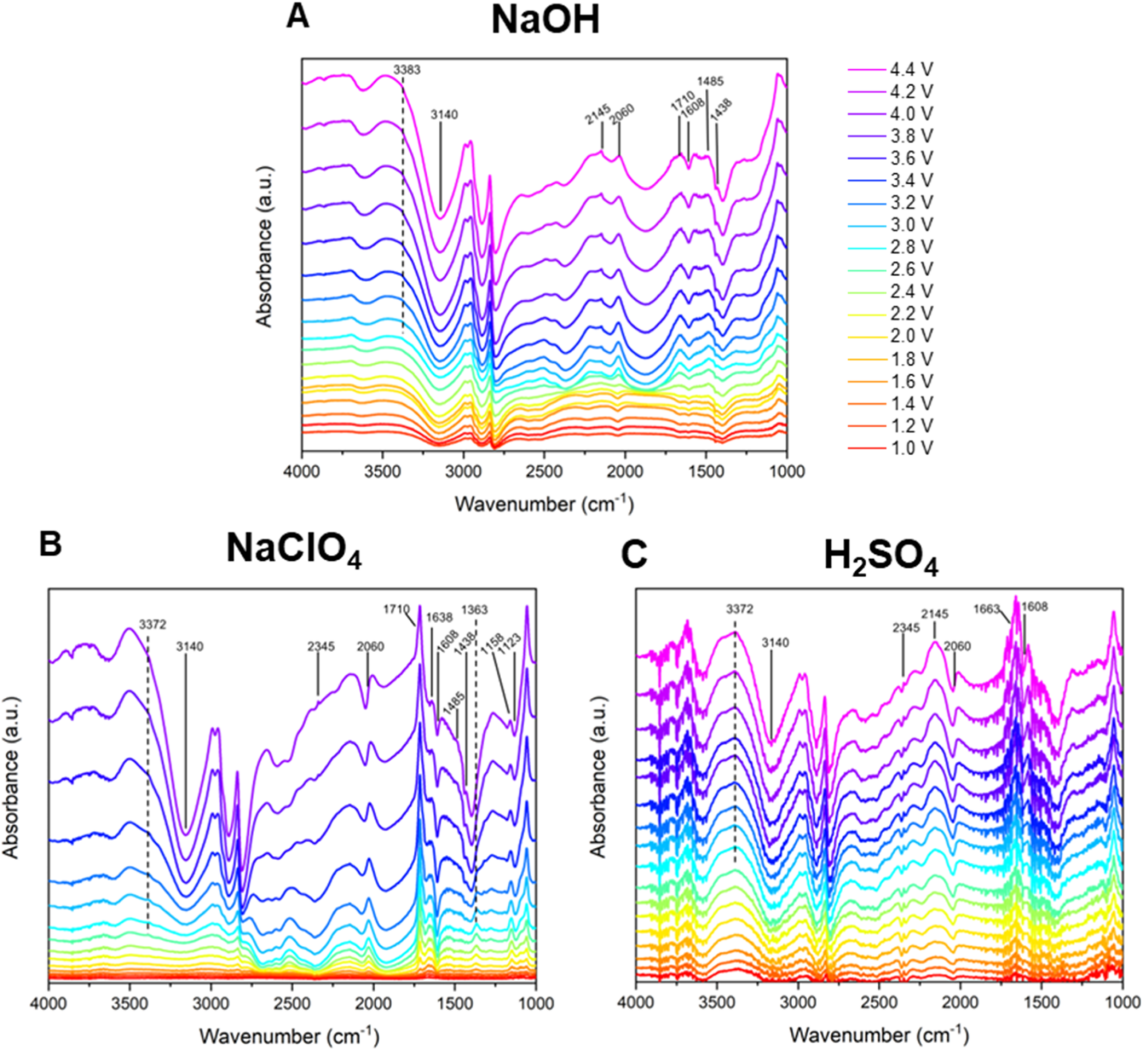
Absorbance FTIR spectra at different potentials vs RHE for the reaction of methylamine in methanol toward methylformamide. Spectra were taken with a GC working electrode in (A) 0.1 M NaOH, (B) 0.1 M NaClO_4_ and (C) 0.05 M H_2_SO_4._ Reference spectra were taken at 1.0 V vs RHE. Reference spectra without the amine are shown in the Supporting Information (SI), Figure S3.

We further studied the mechanism of the reaction with in situ FTIR spectroscopy measurements (Figure 4). Spectra were collected in three different electrolyte conditions, 0.1 M NaOH, 0.1 M NaClO_4_ and 0.05 M H_2_SO_4_. In all cases, we added 0.5 M MeNH_2_ to the electrolytes as the reagent. The spectra were collected with an RHE reference electrode, for which the conversion to SCE is explained in the Experimental Section. Reference spectra without the amine are shown in Figure S3, as well as reference spectra of the starting materials, expected intermediates and products (Figures S4–S8). In the spectra, negative bands are associated with the consumption of a chemical species near or on the electrode surface, while positive bands are associated with the production of intermediates or products. A comparison between spectra taken with (Figure 4) and without MeNH_2_ (Figure S3) shows clear differences: For all electrolytes, methanol oxidation in absence of the amine source shows a large positive band at 2345 cm^–1^, associated with the C–O stretching mode of CO_2_ production.^27^ In NaClO_4_ this band is less obvious, but due to the instability of the thin layer we could not measure at larger potentials than 2.8 V vs RHE in this electrolyte without MeNH_2_. CO_2_ is an expected product, since the full electrochemical oxidation of methanol is a well studied reaction in the field of fuel-cell research.^28−30^ More interestingly, we see that when we add the amine the production of CO_2_ is fully or almost fully inhibited in all electrolytes. Thus, MeNH_2_ inhibits the full oxidation of methanol, possibly by reacting with the intermediates.

Upon addition of MeNH_2_, there are other bands of significance in the FTIR spectra. In all spectra we observe a weak positive band at 3372/3383 cm^–1^, associated with the formation of the N–H bond of formamide.^31,32^ Other positive bands found at 1663/1638 and 1158 cm^–1^ are associated with the C=O and C–O stretching frequencies of formic acid, formed via overoxidation of methanol (Figure S4). Indeed, these bands are absent in NaOH, where formate would be expected, but not formic acid due to the high pH.^33^ At 1363 cm^–1^ the CO_2_^–^ rocking vibration of formate is found, but only in NaClO_4_ (Figure S5).^34^ In H_2_SO_4_ formic acid is in its protonated form due to the low pH. The production of the formaldehyde intermediate is important for the reaction to succeed, but formaldehyde is difficult to identify through IR, mostly because of its presence as dimethylketone in methanol, which does not have the strong C=O stretching frequency.^35^ Nevertheless, we could identify a small positive band at 1485 cm^–1^ associated with the CH_2_ scissoring frequency of formaldehyde.^36^ In the NaClO_4_ electrolyte, there is a strong positive band at 1710 cm^–1^. This could be assigned to the C=O stretching frequency of formic acid (Figure S4), formaldehyde (Figure S6) or the methylformamide product (Figure S7).^32^ In a neutral electrolyte, the formic acid is expected to be deprotonated by methylamine, so only formate would be observed (Figure S5). As mentioned before, formaldehyde is normally present as the dimethylketone in methanol, yet freshly formed formaldehyde might be visible in this electrolyte, even if it is only shortlived. So the band at 1710 cm^–1^ is ascribed to either formaldehyde or the methylformamide product.

Consumption of starting materials is shown in FTIR through negative bands. Methanol consumption is indicated by a negative band at 3140 cm^–1^, corresponding to the O–H stretching frequency.^37^ At 1123 cm^–1^, we also see a negative band corresponding to the C–O–H deformation frequency of methanol.^37^ Consumption of MeNH_2_, which is more evident in NaOH and NaClO_4_ electrolyte, is indicated by negative bands at 1608 and 1438 cm^–1^, associated with the N–H stretching and the CH_3_ antisymmetric deformation, respectively (Figure S8).^38^

The in situ FTIR results show that the oxidation of MeOH in the presence of MeNH_2_ leads to the formation of methylformamide in all electrolytes. The presence of negative bands corresponding to the consumption of both starting materials, as well as the positive bands related to formamide production are clear proof of this. Although only detected in trace amounts after bulk electrolysis, the in situ FTIR results do show the production of formate/formic acid as a side product. Finally, the presence of MeNH_2_ clearly inhibits full MeOH oxidation to CO_2_. While these results are apparent regardless of the used electrolyte, there is a clear difference between the FTIR spectra as discussed below.

When comparing the FTIR spectra in NaClO_4_ with those in NaOH and H_2_SO_4_, the band at 2145 cm^–1^ is missing. In earlier research done on this reaction using ammonia and methanol, this band is associated with a cyanide intermediate,^10,39^ which could form the formamide product upon hydration.^40^ In our research, the band can be ascribed to two different species. Oxidation of methylamine could form cyanide, which would be a side product in this reaction. However, if this frequency is associated with dehydrogenation and dehydration of the C–N bond made from MeNH_2_ and formaldehyde, this would result in the formation of an isocyanide, which also has an IR band between 2100 and 2200 cm^–1^.^41,42^ The band found in our FTIR experiment at 2145 cm^–1^ can thus be associated with both cyanide and methyl isocyanide. The electrochemical synthesis of isocyanides is difficult, until now only one example is published in literature where isocyanides are synthesized oxidatively, requiring an aminotetrazole precursor.^43^ An example using reductive electrolysis has also been reported using carbonimidoyl dichloride precursors.^44^ We are therefore surprised to find them as intermediates in this formamide electrosynthesis. The presence of an isocyanide could also be part of the reason why we do not get a full 100% FE between the formaldehyde and formamide product in Figure 3. Methylisocyanide is known to isomerize to acetonitrile, which is not detected in the HPLC measurements.

**Scheme 2:**
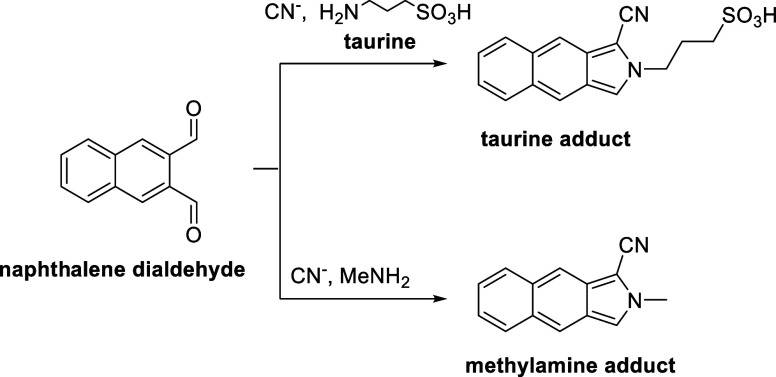
Reaction Pathway for the Cyanide Test with Naphthalene-Dialdehyde in the Presence of Taurine (Top) and Methylamine (Bottom) as the Amine Source

To prove that the bond is associated with an isocyanide, we had to exclude the presence of cyanide from the reaction. We did this by adapting a known procedure for cyanide detection based on the cyclization of naphthalene dialdehyde in the presence of taurine and cyanide (Scheme 2).^23,24^ The resulting product is a *N*–heterocyclic species with a high absorption coefficient, allowing for analysis of cyanide concentrations on a ppm level through HPLC. When testing this method using standard solutions with known cyanide concentrations, the presence of MeNH_2_ shifted the product retention time significantly. We suspected that the large excess of methylamine (the ratio is 10:1) outcompetes taurine in the cyclization reaction, since both are primary amines. This was confirmed by LC-MS measurements (see Figures S9 and S10). We then used the retention time of this methylamine adduct to test for cyanide presence after bulk electrolysis.

To exclude cyanide from the reaction, we performed bulk electrolysis again, with 0.1 M NaOH, 0.5 M MeNH_2_ at 3.0 V vs SCE. Analysis of the bulk electrolysis reactions confirmed that no measurable cyanide was present in the reaction mixture after one hour of electrolysis. Therefore, we assign the band found in the FTIR measurements to methyl isocyanide, which later forms formamide through hydration (See Figure S11).

**Scheme 3:**
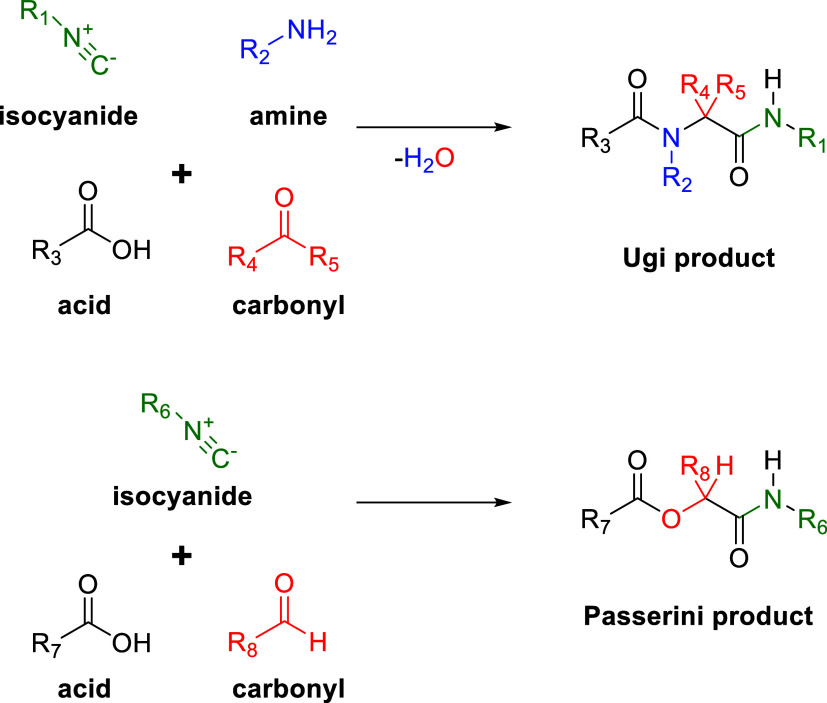
General Reaction Schemes for the Ugi (Top) and Passerini (Bottom) Isocyanide Multicomponent Reactions

To further prove the presence of an isocyanide intermediate in this reaction, we ran a multicomponent reaction involving an isocyanide (IMCR). The two most well-known IMCRs are the Passerini^45^ and Ugi^46^ reactions (Scheme 3). In these reactions, multiple organic reagents come together in a fashion where the product contains most or all of the atoms of the starting materials with no or few side products.^47,48^ The unique amphiphilic nature of isocyanides makes them particularly suitable for MCRs. When looking at the mechanism of the Ugi reaction, this is clearly visible first by the nucleophilic attack of the isocyanide, which then forms a nitrillium ion that is electrophilic and susceptible to follow-up reactions. Because of this unique reactivity, we thought that if a Passerini or Ugi reaction could be performed in the reaction mixture, this would prove that an isocyanide intermediate was present.

**Scheme 4:**
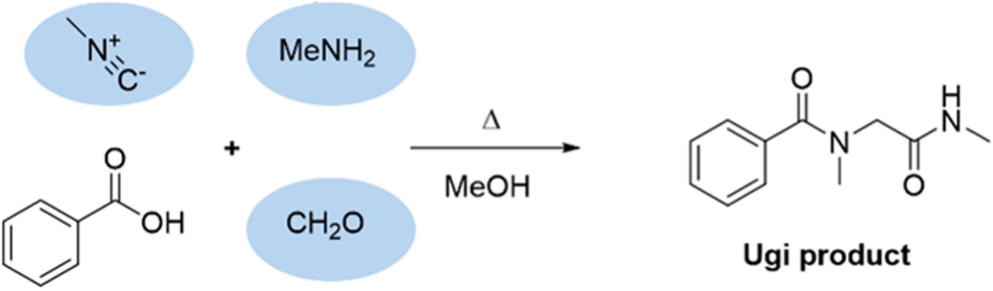
Ugi Reaction Performed with the Reaction Mixture after 24 h Electrolysis at 3 V vs SCE Circled molecules were already present in the reaction mixture after completion of the electrolysis.

Most of the required reagents for an IMCR are already present at the end of the electrochemical reaction. Since no formic acid was detected in our reaction mixtures, a spontaneous IMCR is unlikely. Instead, we decided to add 0.1 M benzoic acid to the reaction mixture after performing electrolysis for 24 h (Scheme 4). The reaction was then placed in an oven and heated at 50 °C for 48 h.^49^ Analysis was performed through HPLC and mass spectrometry. For the HPLC analysis, the Ugi product was synthesized from the methyl ester of Sarcosine (SI Scheme S1). The peak found in the HPLC chromatogram for the synthesized compound matched that of the attempted Ugi reaction. Flow injection analysis electrospray injection mass spectrometry (FIA-ESI-MS) showed the desired protonated molecular ion with a mass of 207.1132 (Figure S12) (calcd. 207.1128, a 1.93 ppm deviation). Combined, this shows that indeed the Ugi reaction was successful and methylisocyanide is an important intermediate in the methylformamide synthesis.

Finally, we studied if there was a preference for the different reaction pathways in the tested electrolytes. The in situ FTIR results show that the electrolyte plays a significant role in the reaction. Dimethylamine was used as the starting material in these experiments, since it cannot form an isocyanide, and is therefore an ideal probe molecule to distinguish between the reaction pathways. Higher FE toward dimethylformamide shows a preference for the dehydrogenation of the hemiaminal, while a lower FE indicates that in that electrolyte, the isocyanide pathway dominates. The results of these electrolysis reactions correspond to the results of the FTIR experiments. In NaOH, the FE to DMF is only 2.1 ± 0.4%. In NaClO_4_ the FE to DMF is 9.2 ± 2.7% and in H_2_SO_4_ it is 0.16 ± 0.01% (Figure S14). In the electrolytes with the isocyanide intermediate, namely NaOH and H_2_SO_4_, the FE for DMF is significantly lower.

**Scheme 5:**
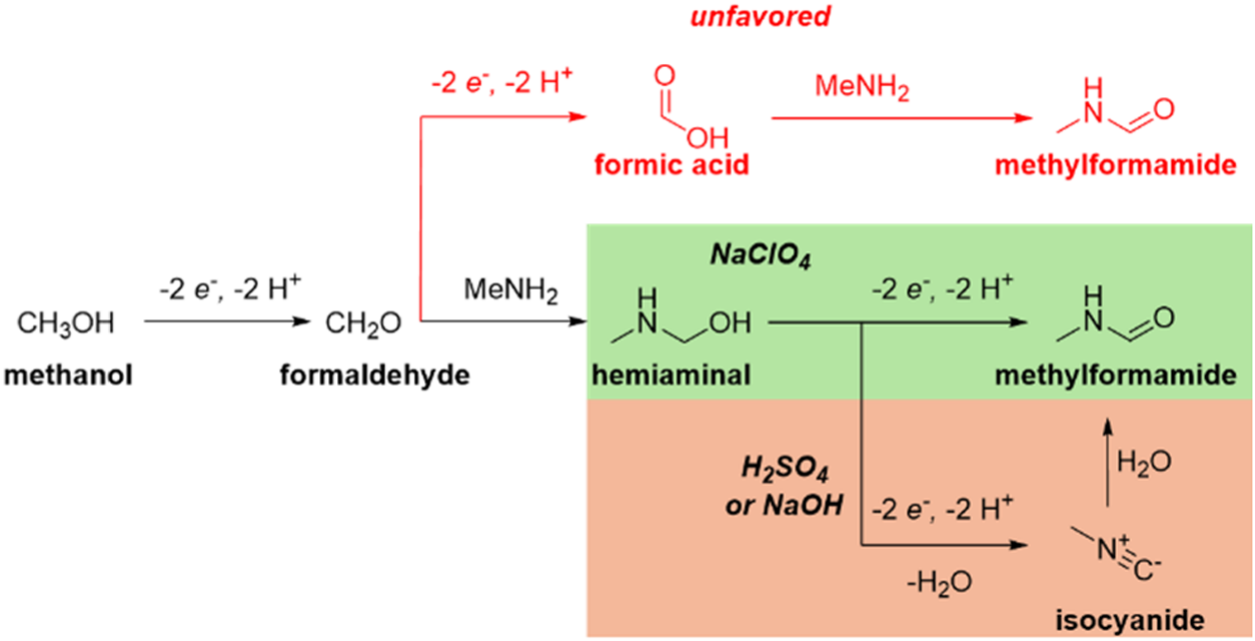
Reaction Pathways for Formamide Electrosynthesis (Top, in Red) via Formic Acid, (Middle) Dehydrogenation and (Bottom) Isocyanide Pathway

**Scheme 6:**
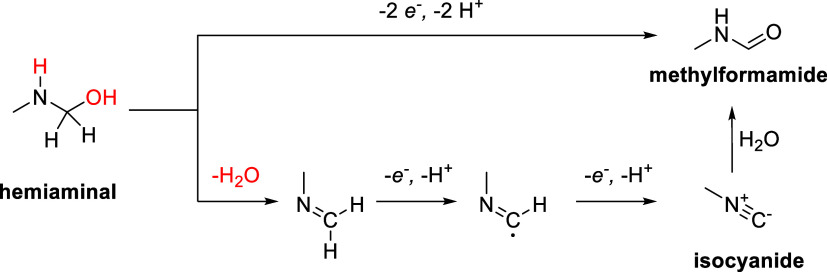
Proposed Mechanism for the Isocyanide Electrosynthesis from the Hemiaminal

The data obtained from bulk electrolysis, spectroscopic analysis and control experiments allows us to propose a mechanism for methylformamide electrosynthesis (Scheme 5). There are three possible pathways: In acidic media, the overoxidation of methanol to formic acid can lead to esterification with methanol, forming methylformate. This will readily react with methylamine in an S_N_2 reaction to give methylformamide. However, since methylformate and formate were only found in trace amounts in our reaction mixtures, this pathway is a minor one in our case. As esterification reactions are typically slow in neutral or basic media, this pathway is unavailable in these electrolytes. The second pathway is the oxidation of the hemiaminal formed from formaldehyde and methylamine. This “direct” oxidation pathway dominates in NaClO_4_. The final pathway is via dehydrogenation and dehydration, where an isocyanide is created, which is later hydrated to form the final methylformamide product. The in situ FTIR results show that this pathway is present in both 0.1 M NaOH and 0.05 M H_2_SO_4_, but not in NaClO_4_. We propose that the formation of the isocyanide intermediate proceeds through a sequential dehydration and dehydrogenation process, as depicted in Scheme 6.^10^ The first step involves dehydration of the hemiaminal, followed by two proton-coupled electron transfer (PCET) steps that lead to the formation of the isocyanide.

This mechanistic hypothesis aligns with previous computational studies that suggest C–N bond oxidation via radical intermediates under electrochemical conditions. Furthermore, our in situ FTIR data confirm the presence of a characteristic isocyanide peak (2145 cm^–1^), which disappears in electrolytes where the radical pathway is not favored. To further validate the isocyanide formation, we conducted a Ugi reaction using the reaction mixture, successfully trapping the isocyanide intermediate.

While this pathway is theoretically plausible, our experimental observations do not support radical involvement as a dominant mechanism. In situ FTIR spectra did not show any radical-related signals, and HPLC analysis of the electrolysis products did not reveal any radical-derived byproducts. Furthermore, if α-amino radicals played a significant role, we would expect dimerization or overoxidation products, but these were not detected under our reaction conditions. These observations indicate that the reaction proceeds through a stepwise dehydration and dehydrogenation mechanism rather than a radical pathway.

### Scope

To investigate the broader applicability of our electrochemical method, we conducted experiments using various aliphatic amines as starting materials (Table 1). Electrolysis was performed in 0.1 M NaOH in methanol at 3.0 V vs SCE, and the faradaic efficiency (FE) for the corresponding formamide products was determined. In addition, to assess whether isocyanides form as intermediates, we conducted extended electrolysis (24 h) and tested for isocyanide presence via HPLC and a subsequent Ugi reaction. Table 1 summarizes the results for the different amine substrates. We observed that ethylamine, isopropylamine, and butylamine undergo electrochemical transformation to their respective formamide products, with isocyanide intermediates confirmed through HPLC and Ugi product analysis. These findings demonstrate that the reaction is not exclusive to methylamine but is applicable to other simple aliphatic amines. However, we also found that functionalized amines such as aminopropionitrile and benzylamine were not compatible under our reaction conditions. In these cases, we observed the formation of a polymer-like film on the electrode surface, likely due to side reactions involving oxidative polymerization. This suggests that while the method is effective for simple aliphatic amines, its compatibility with more functionalized substrates is limited under oxidative electrochemical conditions.

**Table 1:**
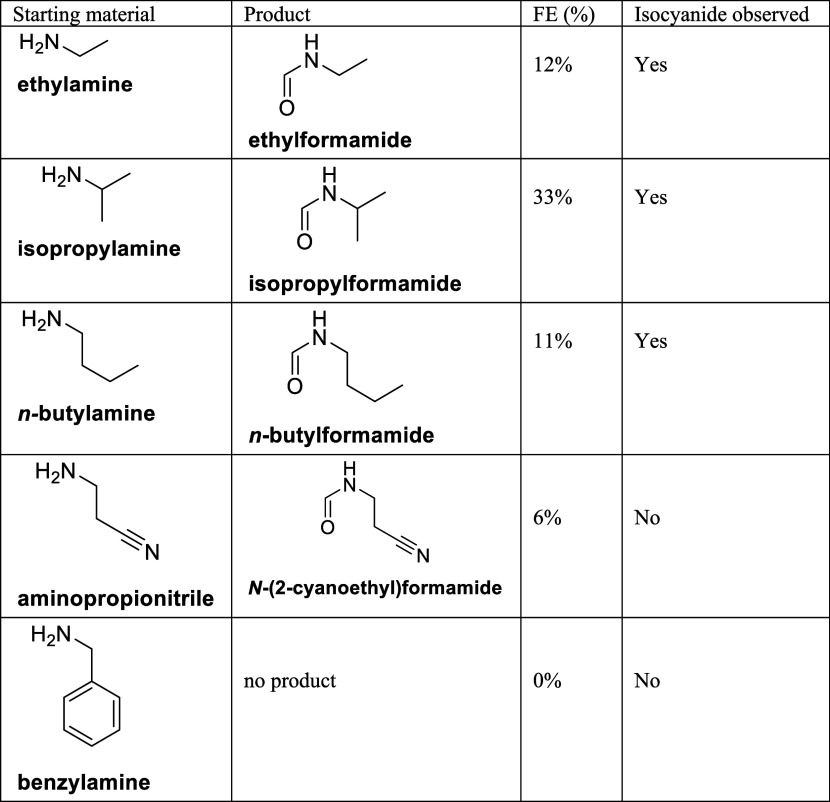
Formamide Synthesis of Different Starting Materials

By extending our study to multiple amine substrates, we confirm that this electrochemical strategy provides a general approach to the synthesis of formamides and isocyanides from aliphatic amines, while also identifying the substrate limitations of the method.

## Conclusions

Here we demonstrate the possibility of electrochemical N-formamide synthesis from methylamine and methanol using a simple glassy carbon electrode, achieving a maximum faradaic efficiency of 34 ± 0.4% for methylformamide in 0.1 M NaClO_4_ at 3 V vs SCE. Mechanistic studies show that there are two main pathways for methylformamide synthesis. Oxidation of the hemiaminal to the formamide is the dominant pathway in NaClO_4_. Oxidation and dehydration to methylisocyanide occurs in H_2_SO_4_ and NaOH, which is later hydrated to methylformamide. The identification of methyl isocyanide as an intermediate, supported by in situ FTIR spectroscopy and further validated through Ugi reaction experiments, highlights the novel capacity of this method to produce isocyanides without complex precursors. We also show that the applicability of the formamide synthesis is not just limited to methylamine, but also extends to other simple aliphatic amines, where isocyanides are still observed as the intermediate.

## Supporting Information Available

In situ FTIR results for methanol oxidation, chromatograms of the cyanide analysis procedure, ^1^H NMR, ^13^C NMR, IR and mass spectrometry results of the Ugi product as well as its synthetic procedure and calibration curves for DNPH-formaldehyde, methylformamide and dimethylformamide analysis (PDF)
