# Supplementary material for: Electrochemical N-Formylation of Amines: Mechanistic Insights and Sustainable Synthesis of Formamides via a Methylisocyanide Intermediate
Source: J Am Chem Soc. 2025 Mar 12;147(12):10298–308. doi: 10.1021/jacs.4c16725 (PMC11951084; doi:10.1021/jacs.4c16725)
Supplement: Supplementary file 1 — ja4c16725_si_001.pdf [file ja4c16725_si_001.pdf]

Supplementary Information

Electrochemical N-Formylation of Amines: Mechanistic Insights and Sustainable Synthesis of Formamides via a Methylisocyanide Intermediate

Pim J.L. Broersen, Lars Wielhouwer, Gadi Rothenberg, Amanda C. Garcia*

Table of Contents

1.0 Calibration methylformamide analysis 2
2.0 Calibration curve for DNPH method for formaldehyde analysis. 3
3.0 FTIR results of methanol oxidation without methylamine 4
4.0 Reference FTIR spectra of starting materials, intermediates and products 5
5.0 Procedure for cyanide analysis 7
6.0 Ugi reaction 9
7.0 Dimethylformamide electrosynthesis and analysis 11
8.0 Synthesis and analysis of different formamides 12
9.0 Mass spectra for Ugi products of different starting materials 13
10.0 Spectroscopic data for synthesized Ugi product from Sarcosine methylester 15

References 17

1.0 Calibration methylformamide analysis

The formamide products were analyzed using a high-performance liquid chromatography (HPLC) Agilent 1260 Infinity II system equipped with an Agilent Technologies Inc. Aminex HPX 87-H column (300×7.8 mm) and an RID and VWD detector. An isocratic chromatography method was used with an eluent of 5 mM H_2SO_4 in water and a column temperature of 65 °C, using a flow rate of 1 mL min^{-1}. The formamide product yield was calculated by fitting the results on a predetermined regression line (Figure S1).

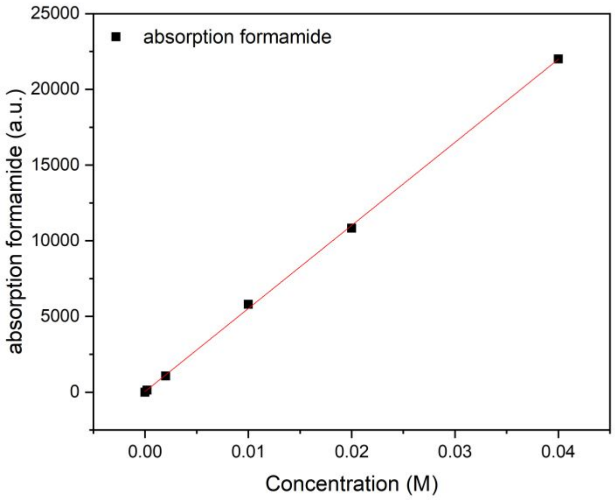

Figure S1. Regression line plot of the HPLC results of standard concentrations of methylformamide against a blank sample. y = 548624x + 40, $R^2 = 0.99$.

2.0 Calibration curve for DNPH method for formaldehyde analysis

We used a modified DNPH derivation method for the analysis of formaldehyde.[2] A 50 µL aliquot was taken from the reaction mixture and diluted 30 times in water. Of this diluted sample, 50 µL was added to a mixture of 400 µL of acetonitrile and 500 µL of water. Then, 50 µL of a 0.3 wt% solution of DNPH in phosphoric acid was added. The sample was heated to 75 °C for 35 min and subsequently analyzed via high-performance liquid chromatography (HPLC). Analysis was done on an Agilent 1260 Infinity II system equipped with an Agilent Technologies Inc. Poroshell 120 EC-C18 column (150 x 3 mm, 2.7µm) and an RID and VWD detector. The column temperature was maintained at 35 °C. The mobile phase was acetonitrile/5 mM H_2SO_4, which was run in a gradient from 20/80 *v/v* to 50/50 *v/v* after 3 min. and 90/10 *v/v* after 8 min. at a flow rate of 1 mL min^{-1}. The formaldehyde derivative was detected via UV at 350 nm and the final concentration was determined by fitting the results to a predetermined regression line (Figure S2).

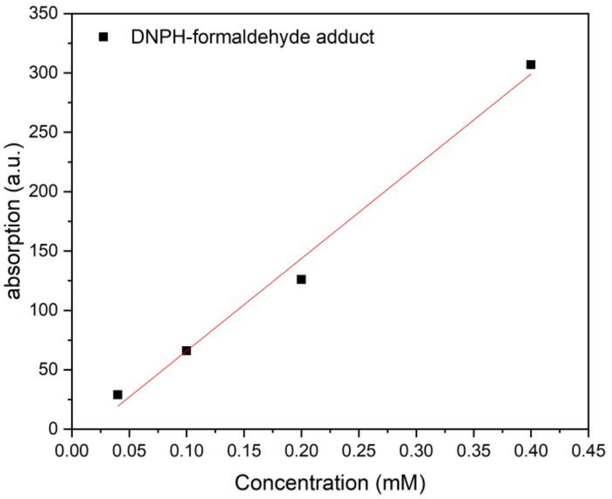

Figure S2. Regression line plot of the HPLC results of DNPH derivation of standard concentrations of formaldehyde against a blank sample. y = 777.5x – 11.8, R^2 = 0.98.

3.0 In situ FTIR results of methanol oxidation without methylamine

As part of the mechanistic studies, in situ FTIR experiments for methanol oxidation without the added amine were performed in 0.1 M NaOH, 0.1 M $NaClO_4$ and 0.05 M H_2SO_4. The results are shown in Figure S3.

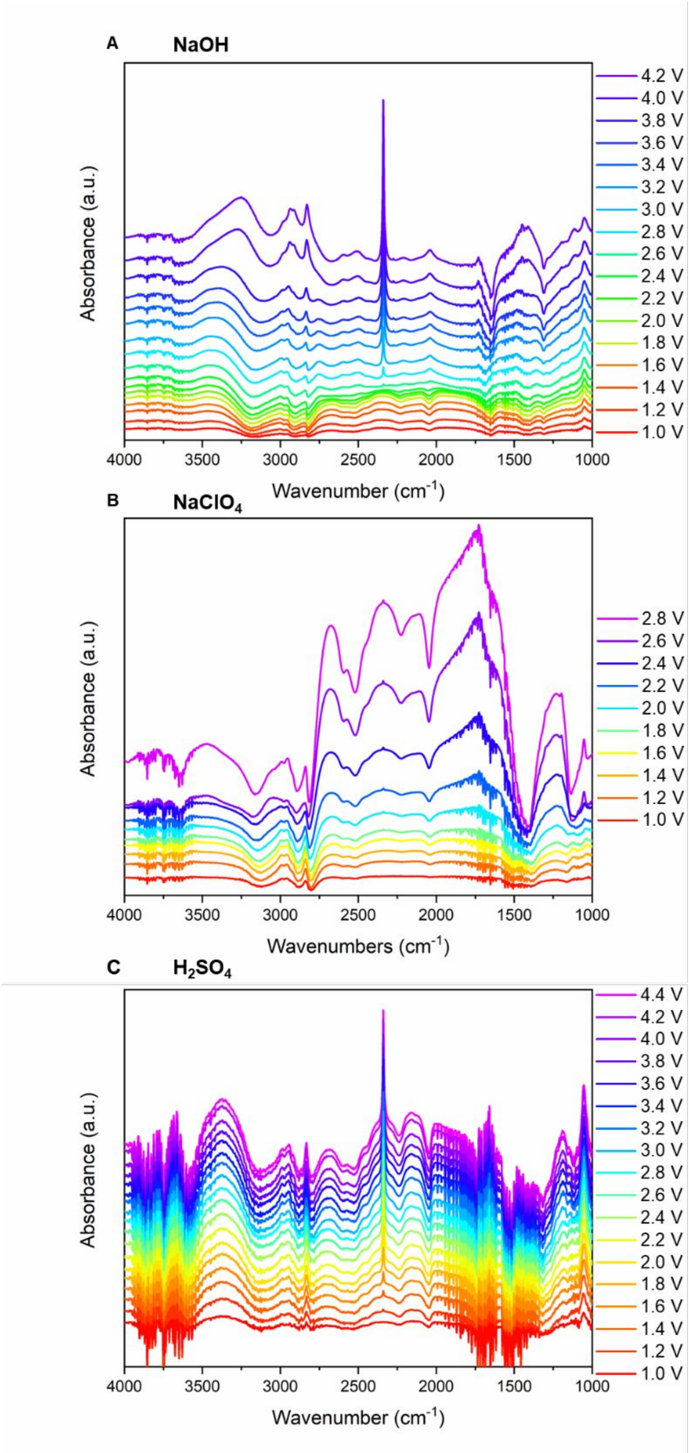

Figure S3. FTIR spectra of MeOH oxidation at different potentials vs. RHE for 0.1 M NaOH (A), 0.1 M $NaClO_4$ (B) and 0.05 M H_2SO_4 (C). A GC working electrode was used with an RHE reference electrode.

4.0 Reference FTIR spectra of starting materials, intermediates and products

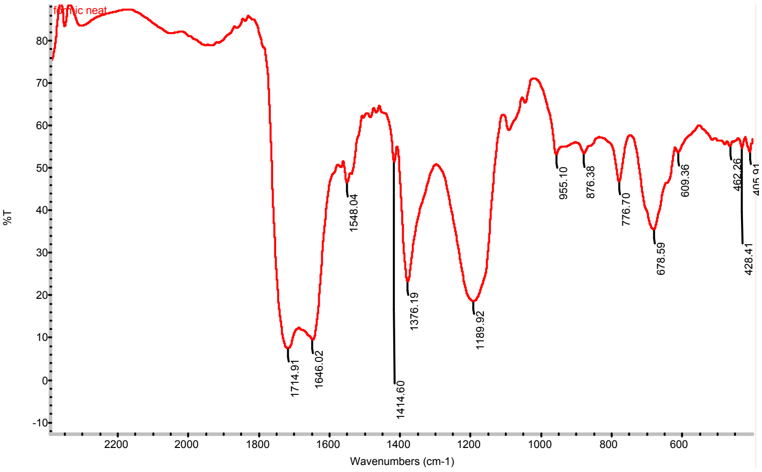

Figure S4. FTIR spectrum of formic acid (neat).

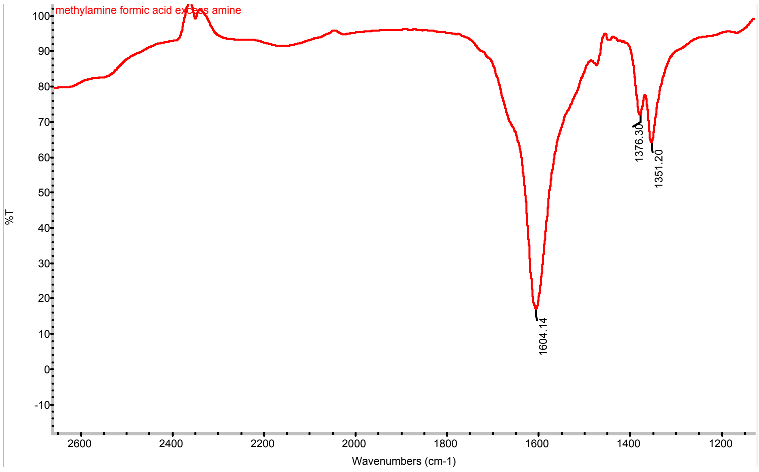

Figure S5. FTIR spectrum of 0.1 M formic acid and 0.5 M methylamine in methanol.

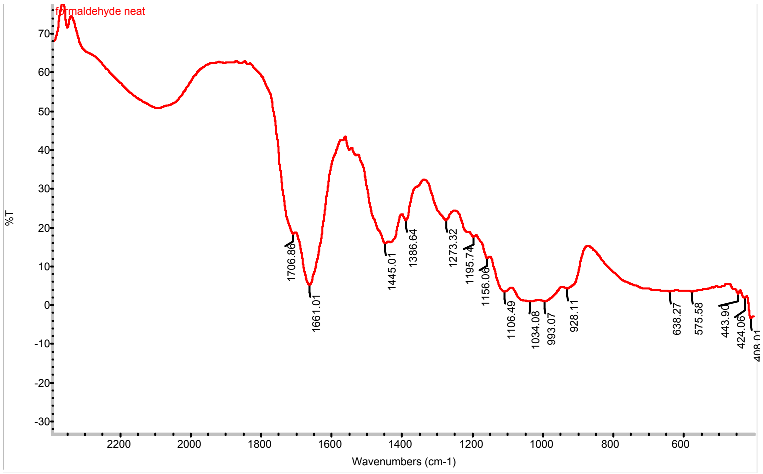

Figure S6. FTIR spectrum of 37% formaldehyde solution, containing 10% methanol as a stabilizer.

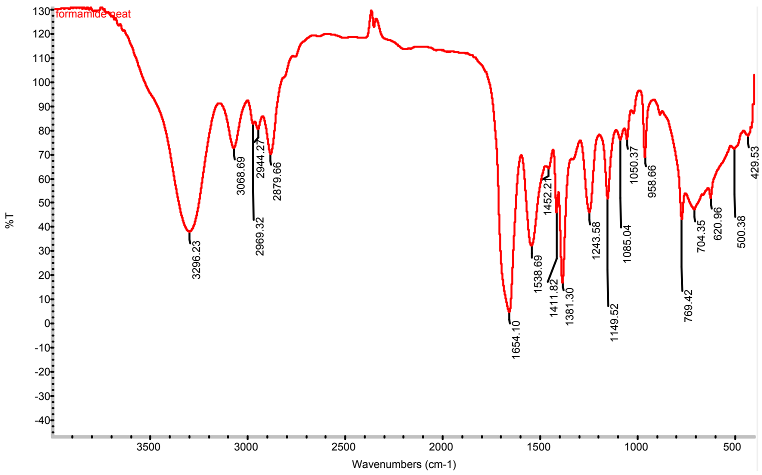

Figure S7. FTIR spectrum of methylformamide (neat).

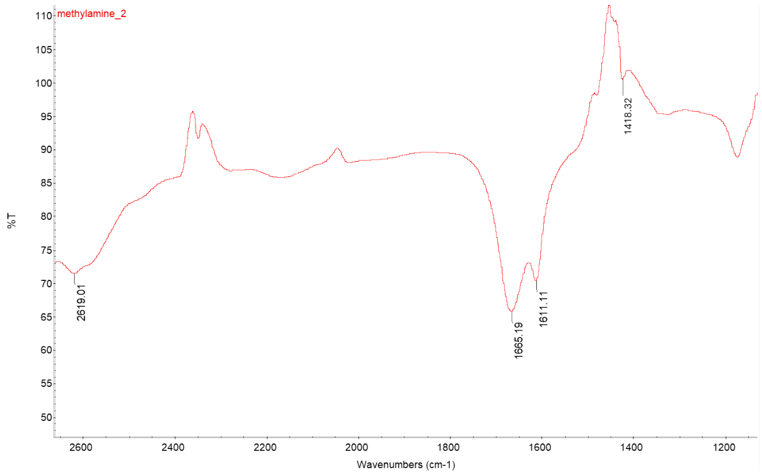

Figure S8. FTIR spectrum of 0.5 M methylamine in methanol.

5.0 Procedure for cyanide analysis

To analyze the cyanide formed in the reaction, we performed control experiments as described in the experimental section of the manuscript. In Figure S9, the chromatogram is shown where methylamine was added to the stock solution of cyanide, NDA and taurine. The methylamine derivative (Main text, Scheme 2) has a retention time of 19.4 min. To confirm that this compound was indeed associated with the methylamine derivative, LC-MS was also performed, of which the chromatogram and associated mass spectrum are shown in Figure S10. This chromatogram shows two peaks, the first peak belongs to the bis-imine, since this is UV active and the LC-MS was measured from 200 to 600 nm. Because this LC-MS experiment was performed on a shorter column, the retention time is different, but the chromatogram peak at 7.816 min. clearly has the mass of the protonated methylamine-cyanide derivative.

Then, we performed bulk electrolysis with 0.1 M NaOH, 0.5 M $MeNH_2$ at 3.0 V vs. SCE for one hour. The cyanide test was performed by taking an aliquot of the reaction mixture, diluting it 10 times in methanol, and adding 125 µL of this mixture to a vial together with 375 µL 0.001 M NaOH in methanol, 100 µL taurine solution, and 100 µL NDA solution. The chromatogram of this mixture is shown in Figure S11. No cyanide derivative is present, proving that no cyanide is formed due to methylamine oxidation under our reaction conditions.

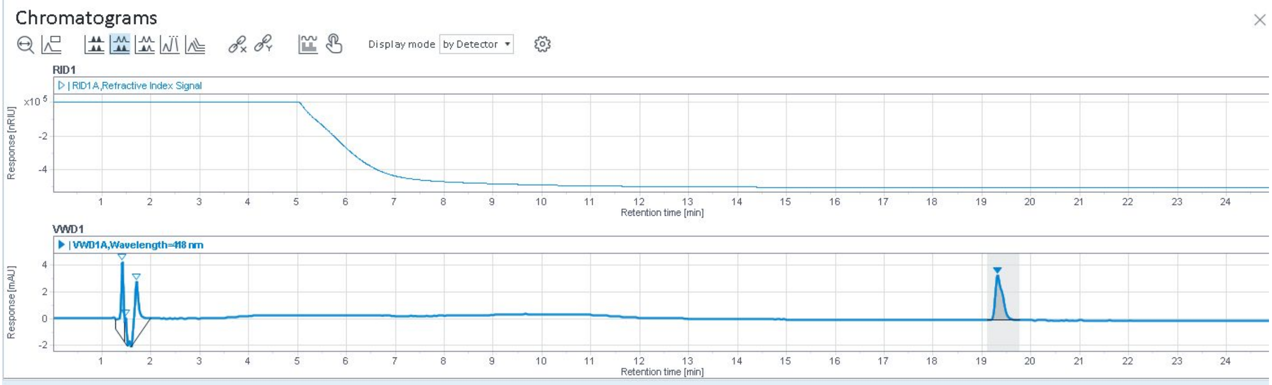

Injection Results

Peaks | Summary

#	Name	Signal description	RT (min)	Area (mAU·s)	Area%	Height (mAU)	Height%	Amount	Concentration	Start time (min)	End time (min)
1		VWD1A,Wavelength=418 nm	1.410	24.149	24.807	5.754	37.17			1.281	1.456
2		VWD1A,Wavelength=418 nm	1.477	4.682	4.810	1.954	12.62			1.456	1.522
3		VWD1A,Wavelength=418 nm	1.698	34.366	35.302	4.369	28.22			1.592	2.004
4		VWD1A,Wavelength=418 nm	19.309	34.151	35.081	3.403	21.98			19.115	19.765

Figure S9. Chromatogram of cyanide adduct with naphthalene-2,3-dicarboxaldehyde. High-performance liquid chromatography was performed on an Agilent Technologies Inc. Poroshell 1260 Infinity II system equipped with an Agilent Technologies Inc. Poroshell 120 EC−C18 column (150x3 mm, 2.7 µm) and an RID and VWD detector. The column temperature was maintained at 35 °C. The mobile phase was acetonitrile/5 mM H_2SO_4, which was run in a gradient from 10/90 v/v to 80/20 v/v after 25 min at a flow rate of 0.5 mL min^{-1}.

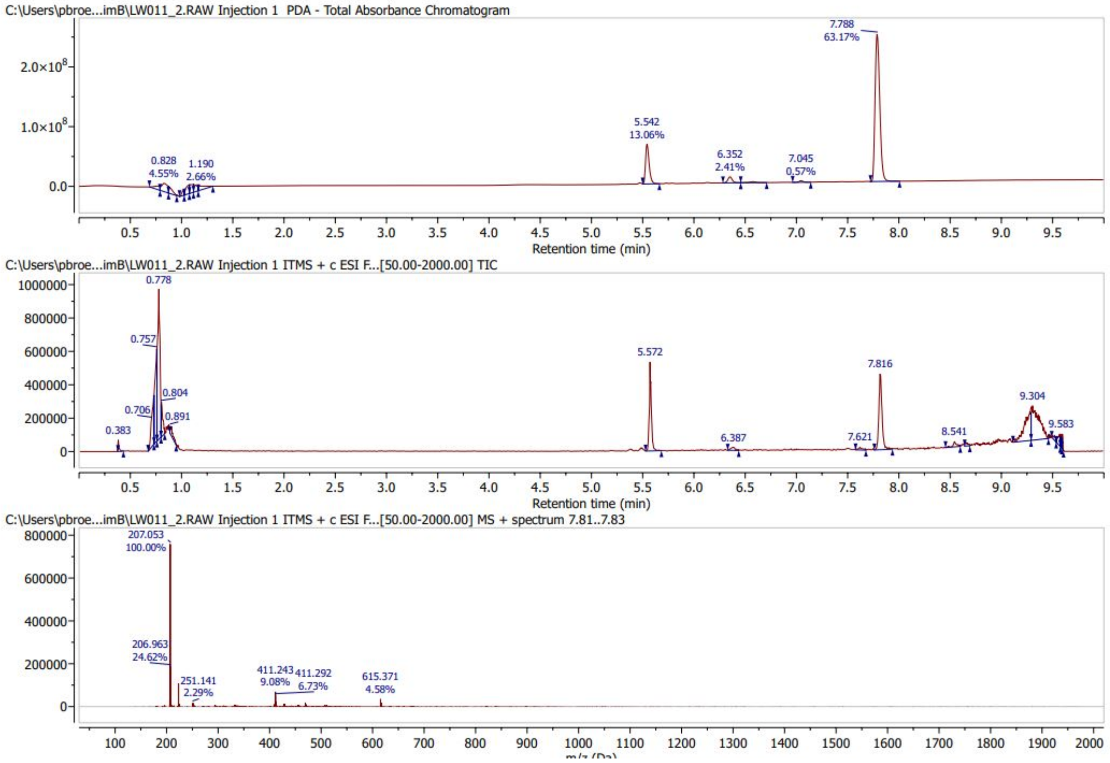

Figure S10. Chromatogram and associated mass spectrum of cyanide adduct with naphthalene-2,3-dicarboxaldehyde. LC-MS was performed on a Shim-pack GIST-HP C18-AQ 3 µm; 100 mm x 4.6 mm (I.D) column, with 0.1% aq. HCOOH/0.1% HCOOH in acetonitrile as the mobile phase. This was run from 95/5 v/v (5 min hold), to 5/95 v/v after 3 min (2 min hold), back down to 95/5 v/v in 1.5 min. (3.5 min. hold). The compounds were detected by UV-Vis from 200-600 nm, The corresponding electrospray ionisation (ESI-Pos) spectra were collected on a HR-ToFBruker Daltonik GmbH (Bremen, Germany) Impact II. The mass spectrum shown in the figure is that of the compound measured at 7.816 min.

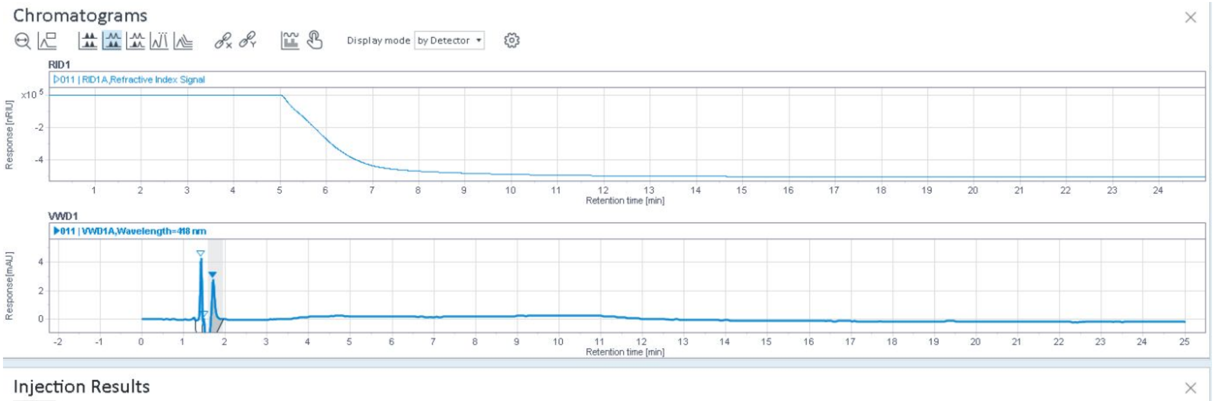

#	Name	Signal description	RT (min)	Area (mAU·s)	Area%	Height (mAU)	Height%	Amount	Concentration	Start time (min)	End time (min)
1		VWD1A,Wavelength=418 nm	1.410	23.462	39.279	5.722	47.96			1.284	1.457
2		VWD1A,Wavelength=418 nm	1.478	4.656	7.795	1.929	16.17			1.457	1.523
3		VWD1A,Wavelength=418 nm	1.699	31.614	52.926	4.280	35.87			1.593	1.961

Figure S11. Chromatogram of the reaction mixture aliquot after adding it to the cyanide testing mixture. Clearly, no cyanide adduct peak is found in the chromatogram. High-performance liquid chromatography was performed on an Agilent Technologies Inc. Poroshell 1260 Infinity II system equipped with an Agilent Technologies Inc. Poroshell 120 EC−C18 column (150x3 mm, 2.7 µm) and an RID and VWD detector. The column temperature was maintained at 35 °C. The mobile phase was acetonitrile/5 mM H_2SO_4, which was run in a gradient from 10/90 v/v to 80/20 v/v after 25 min at a flow rate of 0.5 mL min^{-1}.

6.0 Ugi reaction

The conditions for the Ugi reaction with the electrochemical mixture were as follows: Electrolysis was performed under standard conditions with 0.5 M $MeNH_2$ and 0.1 M NaOH in methanol at 3.0 V vs. SCE for 24 hours. When the electrolysis was finished, a 1 mL aliquot was taken from the reaction and added to a GC vial, along with 13 mg of benzoic acid. This reaction mixture was heated to 50 °C for 48 hours. Analysis was performed with HPLC and mass spectrometry. Both the chromatogram peak and the mass spectrum aligned with the synthesized Ugi product.

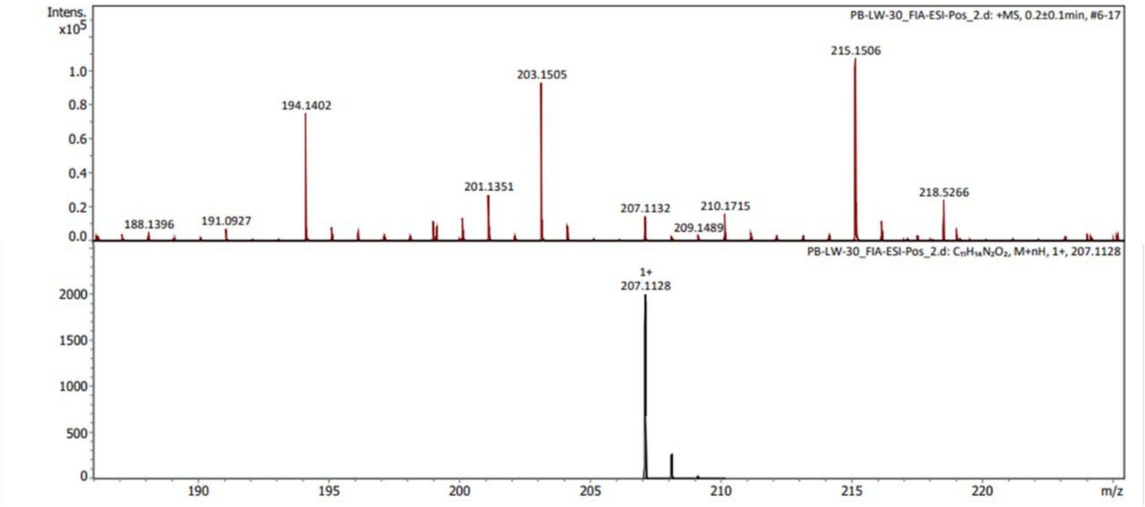

Figure S12. Mass spectrum of the Ugi product mixture (top) and simulated mass of the Ugi product ($M+H^+$) (bottom).

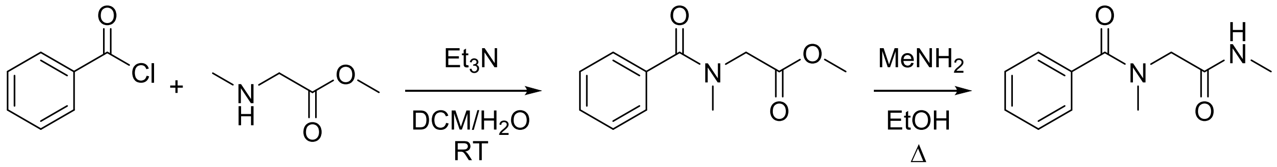

Scheme S1. Procedure for the synthesis of the Ugi product from Sarcosine methylester.

The desired Ugi product was also synthesized from the methyl ester of Sarcosine through the following method (Scheme S1): Sarcosine methyl ester hydrochloride (1 g, 7.16 mmol) and triethylamine (2.2 mL, 15.75 mmol) were dispersed in a minimum amount of H_2O (5 mL) and CH_2Cl_2 (10 mL) followed by dropwise addition of benzoyl chloride (1.25 mL, 10.74 mmol) at 0 °C. The resulting mixture was stirred at room temperature for 3 hours. 10 mL of brine was added and the organic layer was collected. The aqueous was extracted with 2×10 mL EtOAc, the combined organic layers were dried over $MgSO_4$ and the solvent was evaporated under reduced pressure. Rf = 0.25 (2/1 CyHex/EtOAc) Subsequently, flash column chromatography was performed with a v/v CyHex/EtOAc gradient of 1/0 to 7/3 to obtain the purified product in a 2/1 rotameric mixture with a yield of 59%. Spectroscopic data agreed with that found in literature.[1]

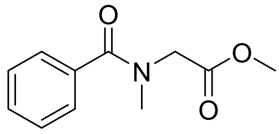

methyl *N*-benzoyl-*N*-methylglycinate

Rf = 0.25 (33% EtOAc/CyHex). ^1H NMR (400 MHz, $CDCl_3$) δ 7.55 – 7.31 (m, 5H), 4.29 (s, 1.3H), 4.00 (s, 0.7H), 3.79 (s, 2H), 3.75 (s, 1H), 3.12 (s, 1H), 3.04 (s, 2H).

An ethanol gas trap was set up by bubbling N_2 gas through methylamine (40% aq. Solution, 100 mL) at room temperature. The methylamine enriched gas was then bubbled through 3 mL of ethanol at 0 °C until the volume had increased by 1.5 mL. This mixture was transferred to a pressure tube, along with 200 mg of the methyl *N*-benzoyl-*N*-methylglycinate. The pressure tube was capped and the reaction mixture was heated to 80 °C for 18 hours. The reaction mixture was allowed to cool to room temperature and the solvent was evaporated under reduced pressure. The pure final product was obtained without additional purification in a 3/1 rotameric ratio with a yield of 64%.

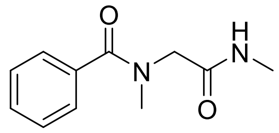

***N*-methyl-*N*-(2-(methylamino)-2-oxoethyl)benzamide**

^{1}H NMR (300 MHz, $CDCl_3$) δ 7.43 (s, 5H), 4.14 (s, 1.5H), 3.92 (s, 0.5H), 3.09 (s, 3H), 2.82 (s, 3H); ^{13}C NMR (75 MHz, $CDCl_3$) δ 130.37 (CH), 128.55 (CH), 127.37 (CH), 52.44 (CH_2), 39.20 (CH_3), 26.32 (CH_3); IR (neat): νmax (cm^{-1}) = 3309 (w), 1659 (w), 1620 (s), 1601 (w), 1563 (w), 1501 (w), 1467 (w), 1447 (w), 1403 (m), 1364 (w), 1255 (w), 1159 (w), 1088 (w), 1051 (w), 1030 (w), 971 (w), 901 (w), 783 (m), 695 (s), 652 (m), 577 (w), 552 (m), 410 (w); HRMS (ESI): m/z calculated for $C_{11}H_{14}N_2O_2$ $[M+H]^+$ 207.1134, found: 207.1135

7.0 Dimethylformamide electrosynthesis and analysis

We studied if there was a preference for the different reaction pathways in the tested electrolytes. This was tested by performing electrolysis reactions with 0.5 M dimethylamine at 3.0 V vs. SCE for 1 hour. The electrolysis was performed in 0.1 M NaOH, 0.1 M $NaClO_4$ and 0.05 M H_2SO_4. The conditions were otherwise identical to those described in the bulk electrolysis section of the experimental section. Analysis of the reaction mixture was performed via HPLC, with the same method as for the methylformamide analysis. Under these conditions, dimethylformamide has a retention time of 28.4 minutes. The dimethylformamide yield was determined by fitting the results to a predetermined regression line (Figure S13). In NaOH, the FE to DMF is only 2.1±0.4%. In $NaClO_4$ the FE to DMF is 9.2±2.7% and in H_2SO_4 it is 0.16±0.01% (Figure S14).

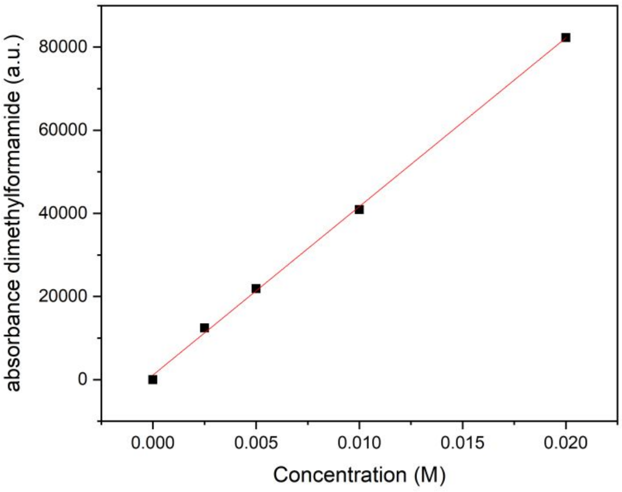

Figure S13. Regression line plot of the HPLC results of standard concentrations of dimethylformamide against a blank sample. y = 4056247x + 1086, R^2 = 0.99.

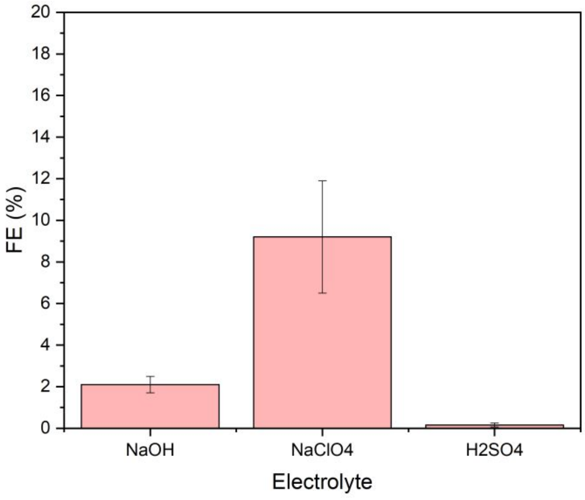

Figure S14. Faradaic efficiency optimization studies of dimethylformamide electrosynthesis varying the electrolyte. Standard conditions for the reaction were 0.5 M $MeNH_2$, 0.1 M NaOH in methanol, using a GC WE, graphite CE applying a potential of 3.0 V vs. SCE for 1 h in a divided cell setup.

8.0 Synthesis and analysis of different formamides

Ethyl formamide was available commercially. To synthesize the other formamides, the following procedure was followed[3]: Amine was mixed with ethyl formate (1.25 M) and heated to reflux for 16 h. The excess ethyl formate was concentrated in vacuo to obtain the pure formamide without further purification. The formamide reference compounds were used to construct calibration curves using the same HPLC method as for the methylformamide analysis

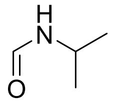

***N*-isopropylformamide**

4/1 rotameric mixture: ^{1}H NMR (300 MHz, CDCl3) δ 8.17 (s, 1H), 4.19 (h, J = 6.8 Hz, 0.8H), 3.75 (h, J = 6.8 Hz, 0.2H), 1.25 (d, J = 6.8 Hz, 6H).

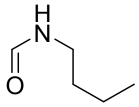

***N*-butylformamide**

5/1 rotameric mixture: ^{1}H NMR (300 MHz, $CDCl_3$) δ 8.18 (s, 0.83H), 8.04 (d, *J* = 12.0 Hz, 0.17H), 3.32 (q, *J* = 6.7 Hz, 1.66H), 3.23 (q, *J* = 6.7 Hz, 0.34H), 1.59 – 1.46 (m, 2H), 1.44 – 1.27 (m, 2H), 0.94 (t, *J* = 7.2 Hz, 3H).

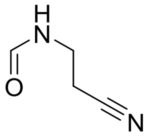

***N*-(2-cyanoethyl)formamide**

20/1 rotameric mixture: ^{1}H NMR (300 MHz, $CDCl_3$) δ 8.22 (s, 1H), 8.13 (d, *J* = 11.7 Hz, 0.06H), 3.56 (q, *J* = 6.3 Hz, 2H), 2.66 (t, *J* = 6.3 Hz, 2H).

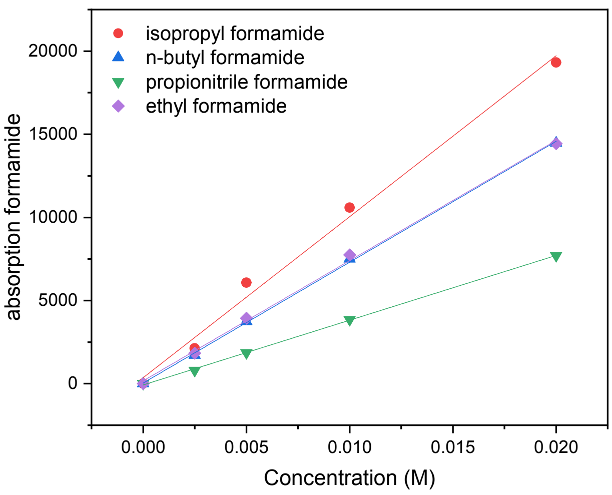

Figure S15. Regression line plot of the HPLC results of standard concentrations of different formamides against a blank sample. Isopropylformamide y = 968210x + 357, R^2 = 0.992, *n*-butylformamide y = 726890x + 34, R^2 = 0.999, propionitrileformamide y = 389490x – 78, R^2 = 0.999, ethylformamide y = 723234x + 166, R^2 = 0.998.

9.0 Mass spectra for Ugi products of different starting materials

The reaction conditions for the Ugi reaction were the same as in SI section 6, using ethylamine, isopropylamine and butylamine as starting materials (Figure S16-S18).

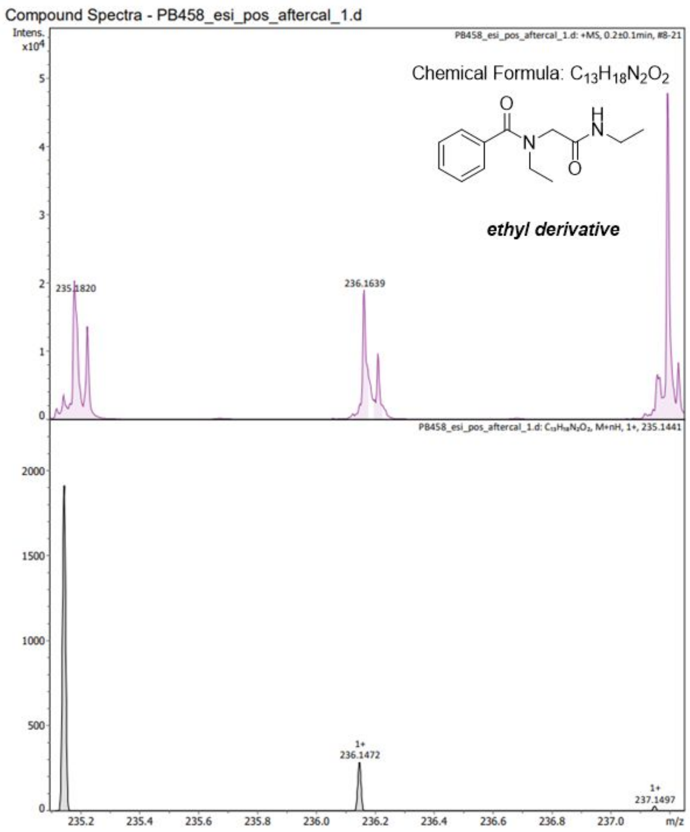

Figure S16. Mass spectrum of the Ugi product mixture (top) and simulated mass of the Ugi product (M+H$^+$) (bottom) of ethylisocyanide.

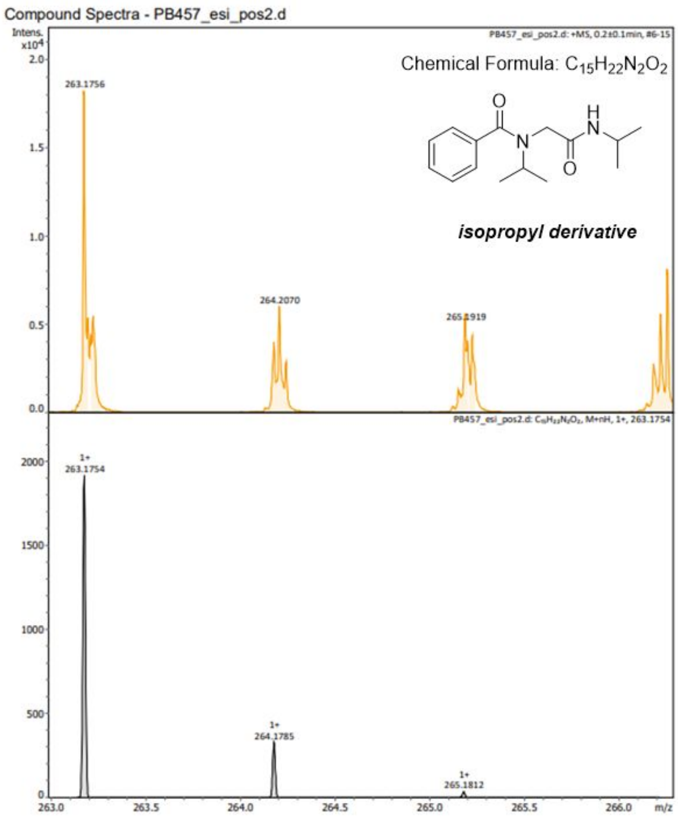

Figure S17. Mass spectrum of the Ugi product mixture (top) and simulated mass of the Ugi product (M+H$^+$) (bottom) of isopropylisocyanide.

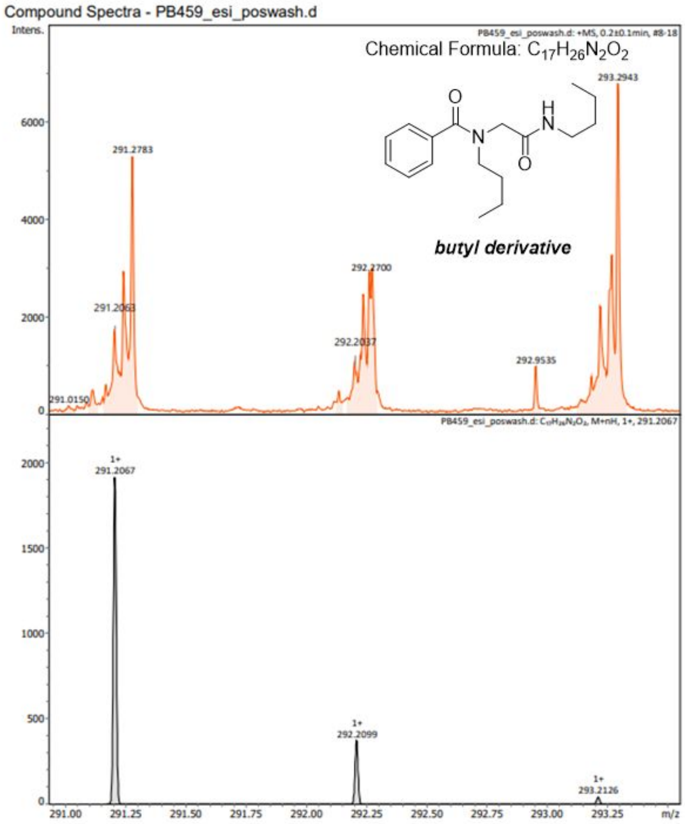

Figure S18. Mass spectrum of the Ugi product mixture (top) and simulated mass of the Ugi product (M+H$^+$) (bottom) of butylisocyanide.

10.0 Spectroscopic data for synthesized Ugi product from Sarcosine methylester

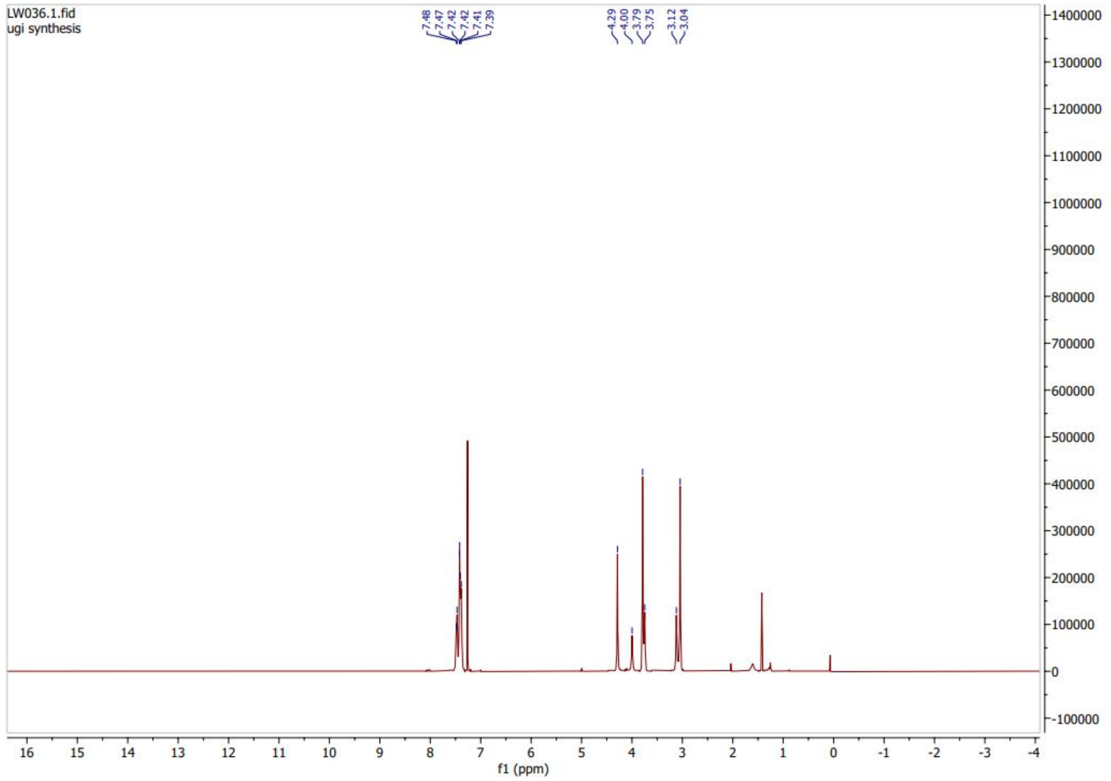

Figure S19. 400 MHz ^{1}H-NMR spectrum of methyl N-benzoyl-N-methylglycinate in deuterated chloroform.

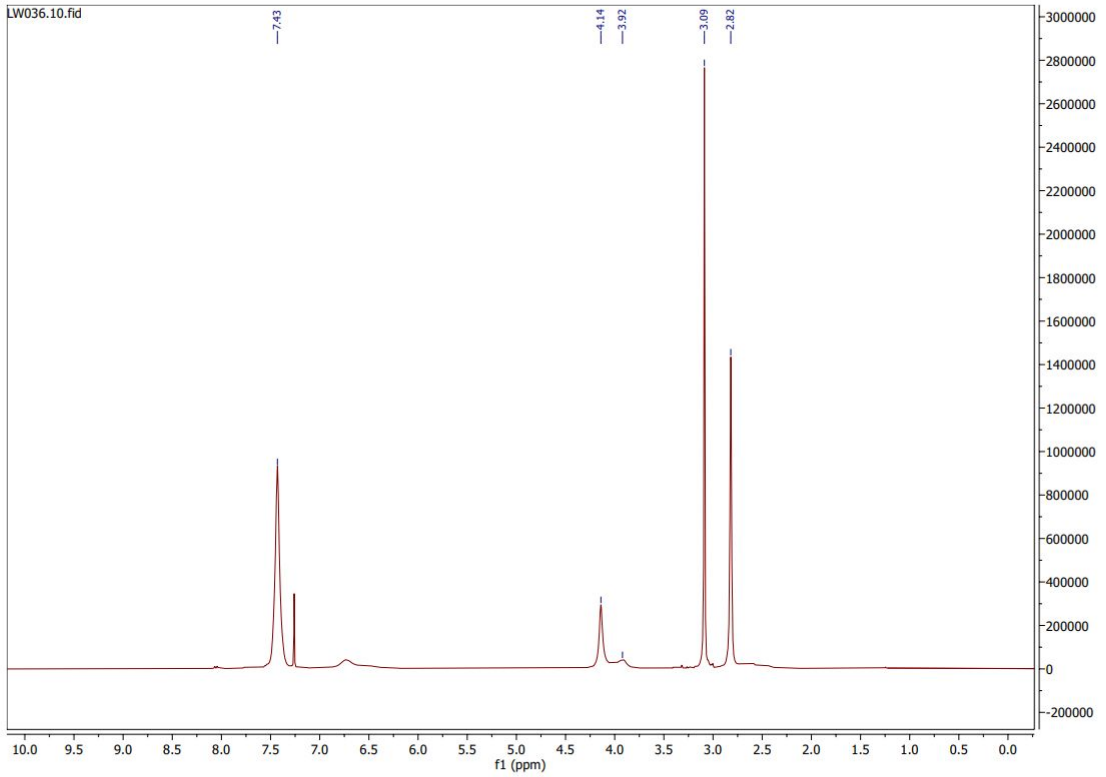

Figure S20. 300 MHz ^{1}H-NMR spectrum of N-methyl-N-(2-(methylamino)-2-oxoethyl)benzamide in deuterated chloroform.

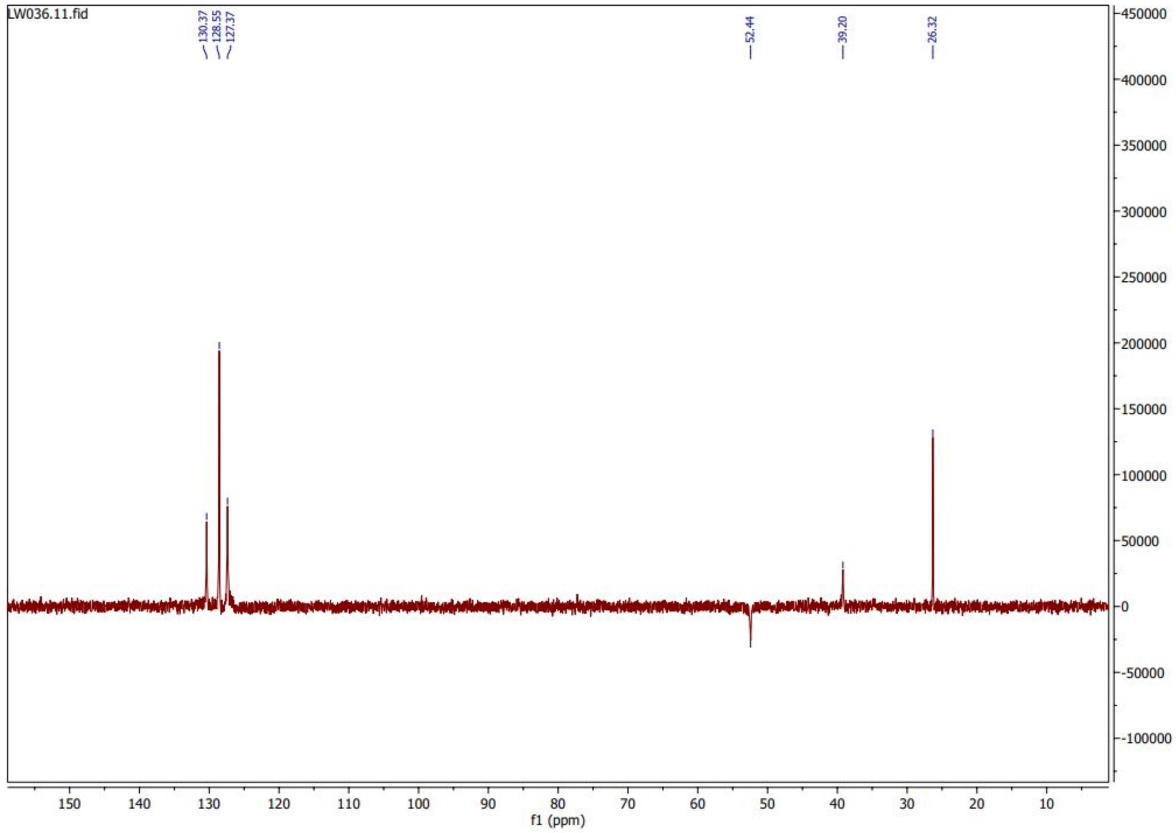

Figure S21. 75 MHz ^{13}C-NMR spectrum of N-methyl-N-(2-(methylamino)-2-oxoethyl)benzamide in deuterated chloroform.

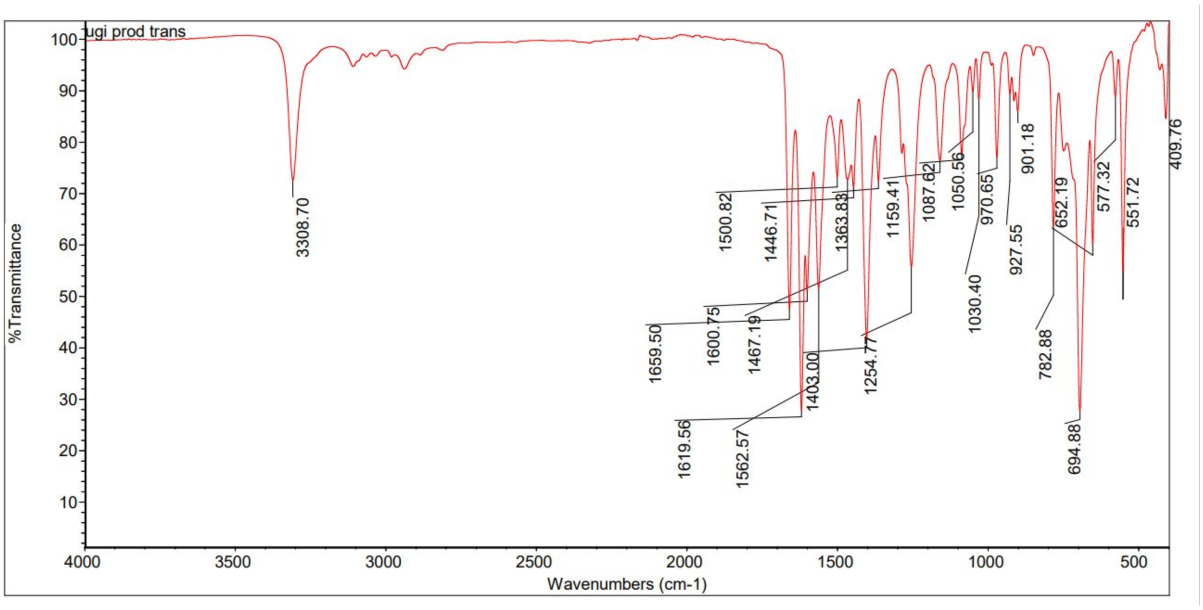

Figure S22. Transmission infra-red spectrum of neat N-methyl-N-(2-(methylamino)-2-oxoethyl)benzamide.

References

(1) Venugopal, N.; Moser, J.; Vojtíčková, M.; Císařová, I.; König, B.; Jahn, U. Single Electron Transfer-Induced Selective α-Oxygenation of Glycine Derivatives. *Adv. Synth. Catal.* **2022**, *364* (2), 405–412. https://doi.org/10.1002/ADSC.202100964.

(2) Bourgeois, C.; Blanc, N.; Cannot, J. C.; Demesmay, C. Towards a Non-Biased Formaldehyde Quantification in Leather: New Derivatization Conditions before HPLC Analysis of 2,4-Dinitrophenylhydrazine Derivatives. *Mol.* **2020**, *25* (23), 5765. https://doi.org/10.3390/MOLECULES25235765.

(3) Saya, J. M.; Oppelaar, B.; Cioc, R. C.; Van Der Heijden, G.; Vande Velde, C. M. L.; Orru, R. V. A.; Ruijter, E. Synthesis of Polycyclic Spiroindolines by Highly Diastereoselective Interrupted Ugi Cascade Reactions of 3-(2-Isocyanoethyl)Indoles. Chem. Commun. 2016, 52 (84), 12482–12485. https://doi.org/10.1039/C6CC07459F.
